# Advances and Challenges in Plant Sterol Research: Fundamentals, Analysis, Applications and Production

**DOI:** 10.3390/molecules28186526

**Published:** 2023-09-08

**Authors:** Dmitry D. Evtyugin, Dmitry V. Evtuguin, Susana Casal, Maria Rosário Domingues

**Affiliations:** 1 CICECO, University of Aveiro, Campus Universitário de Santiago, 3810-193 Aveiro, Portugal; dmitry.evtyugin@ua.pt (D.D.E.); dmitrye@ua.pt (D.V.E.); 2 LAQV-REQUIMTE, Faculty of Pharmacy, University of Porto, 4050-313 Porto, Portugal; 3 Mass Spectrometry Centre, LAQV-REQUIMTE, Department of Chemistry, University of Aveiro, Campus Universitário de Santiago, 3810-193 Aveiro, Portugal; 4 CESAM, Centre for Environmental and Marine Studies, Department of Chemistry, University of Aveiro, Campus Universitário de Santiago, 3810-193 Aveiro, Portugal

**Keywords:** phytosterols, oxidation products, sitosterol, analysis, production, biosynthesis

## Abstract

Plant sterols (PS) are cholesterol-like terpenoids widely spread in the kingdom Plantae. Being the target of extensive research for more than a century, PS have topped with evidence of having beneficial effects in healthy subjects and applications in food, cosmetic and pharmaceutical industries. However, many gaps in several fields of PS’s research still hinder their widespread practical applications. In fact, many of the mechanisms associated with PS supplementation and their health benefits are still not fully elucidated. Furthermore, compared to cholesterol data, many complex PS chemical structures still need to be fully characterized, especially in oxidized PS. On the other hand, PS molecules have also been the focus of structural modifications for applications in diverse areas, including not only the above-mentioned but also in e.g., drug delivery systems or alternative matrixes for functional foods and fats. All the identified drawbacks are also superimposed by the need of new PS sources and technologies for their isolation and purification, taking into account increased environmental and sustainability concerns. Accordingly, current and future trends in PS research warrant discussion.

## 1. Introduction

Mammalian and fungal cells typically have one main sterol specie in the lipid pool, while plants contain many complex cholesterol-like structures that gain the common name of phytosterols (PS). Among them, sitosterol, first described by Richard Burián in 1897 (who named the compound from wheat germ oil after its origins: from sitos, meaning “grain” in Greek) [1] and finally characterized by works of Rudolph John Anderson in 1926 [2,3], is the most representative molecule of this class of lipids in plants and can be seen as the plant counterpart of mammalian cholesterol. In fact, it was the parallel works on cholesterol and bile acids, in the span of more than a century, that culminated in the definite structural elucidation of the sterol molecule in 1932, propelling the progress in natural products chemistry [4] and bringing new insights on PS, whose metabolism and absorption were already being discussed at the time [5]. Nonetheless, it was only in 1951 that PS formulations, with sitosterol as the main component, were described for the first time as a therapeutic agent for hypercholesterolaemia [6]. In the 1950s the first PS-based drug, Cytellin [7], was marketed in the United States as cholesterol-lowering pharmaceutical by Eli Lilly Company and ended up being prescribed for more than 20 years with a remarkable safety record [8]. With the introduction of fibrates in the 60s and statins in the 80s [9] one would expect that plant sterols would start to have a secondary role, but since the 50s hundreds of clinical trials have been conducted to prove PS effectiveness and safety in lowering serum total and low-density lipoprotein cholesterol, not to mention the studies on other applications (e.g., benign prostate hyperplasia and androgenetic alopecia with sitosterol-based products) [10,11]. As a result, PS products still surmounted with new formulations and after the first PS functional food, Benecol^®^, was launched by Rasio Oy in Finland (1995), in the next five years PS products have been introduced in more than 20 countries [12,13].

Aside from the trendy hypocholestrolemic effect, steroid-drug chemical synthesis starting from PS has also been a paradigmatic application of PS since the discovery of a precipitated mixture of stigmasterol and sitosterol in soybean oil tanks by Percy Julian in 1939. This has enabled a steady separation process for production of stigmasterol in the 40s [14]. The later sterol having a double bond in the side chain was one of the most important steroid-starting materials for commercial production of the female hormone, progesterone [14,15]. Microbial biotransformation of PS has been recognised since the 50s and has been gaining importance in pharmaceutical industry to produce steroid-drugs (e.g., sex hormones, corticosteroids, etc.) [16,17]. Finally, PS relevance is also implied in several patents of PS-based products [18,19,20,21,22,23,24,25,26,27,28] and PS production-related methodologies [29,30,31,32,33,34,35,36,37,38,39,40,41]. This review intends to outline a comprehensive overview of fundamental and applied PS research leading to further development in this field and prompt new approaches in commercial exploitation. PS research is also directly related to the general evolution and development of relevant analytical methodologies, which are also discussed in this review article.

## 2. Structures, Biosynthesis and Function

### 2.1. Sterol Nomenclature and Structures

**Figure 1:**
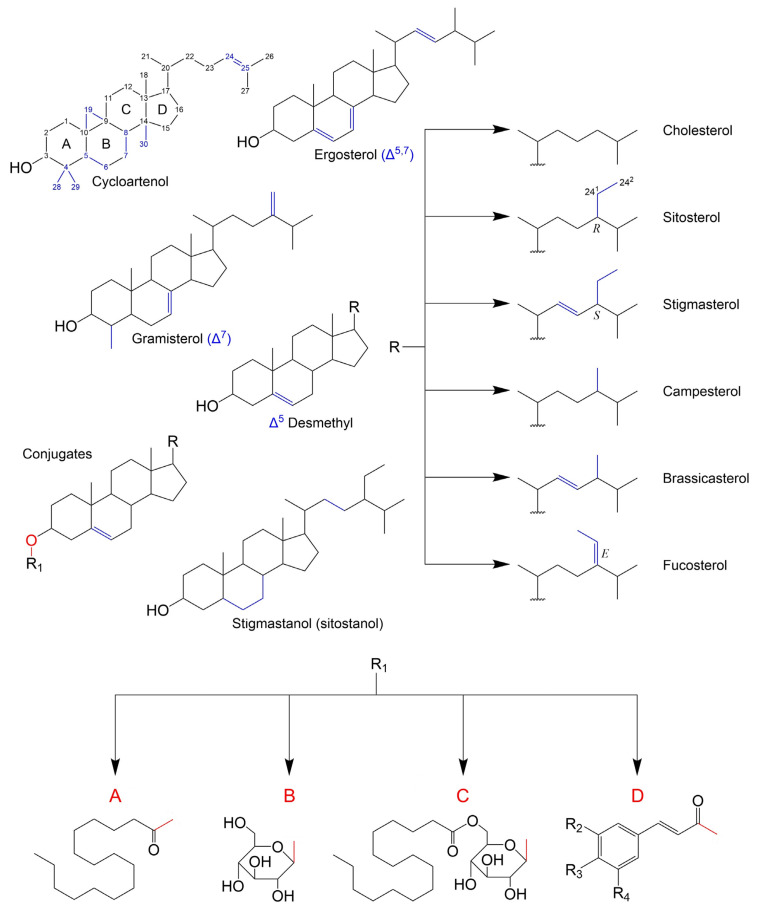
Structures of existing classes of free PS and PS conjugates. Letters A, B and C correspond to steryl palmitate, steryl β-d-glucoside and steryl palmitoyl-β-d-glucoside, respectively. Letter D refers to steryl esters with different possible phenolic acid moieties, being coumarate: R_2_-H, R_3_-OH and R_4_-H; caffeate: R_2_-OH, R_3_-OH and R_4_-H; ferulate: R_2_-OCH_3_, R_3_-OH and R_4_-H; cinnamate: R_2_-H, R_3_-H and R_4_-H; and sinapate: R_2_-OCH_3_, R_3_-OH and R_4_-OCH_3_. Characteristic structural features are marked in blue.

Based on biosynthetic origins and specific stereochemistry, a sterol can be defined as a chiral tetracyclic isoprenoid which is normally formed by cyclization of squalene oxide through the transition state possessing stereochemistry similar to the *trans-syn-trans-anti-trans-anti* configuration and retains a polar group at C-3 (hydroxyl or keto), an *all*-*trans-anti* stereochemistry in the ring-system and a side chain 20*R*-configuration [42] (pp. 1–40). As a consensus, sterols are differentiated by variations within the cyclopentanoperhydrophenanthrene ring system and/or sidechain modifications, being the carbon numbering and some PS examples presented in Figure 1. In terms of structural and biosynthetic grounds, PS are divided into: 4,4-dimethylsterols (e.g., lanosterol), which retain both methyl groups at C-4; the 4α-(mono)methylsterols (e.g., gramisterol), which retain a methyl group at the C-4α position; and the 4-desmethyl (e.g., sitosterol), from which both methyl groups at C-4 have been removed. Both 4-dimethyl and 4α-methylsterols are precursors of 4-desmethylsterols and exist at lower concentrations. The latter are also categorized into Δ^5^-sterols, Δ^7^-sterols and Δ^5,7^-sterols according to the position and number of double bonds in the B ring. Lastly fully saturated counterparts of sterols are named stanols (e.g., sitostanol). Another distinction is made according to side chain alkylation at the C24 position, which will also reflect on the total number of carbons in the molecule. As such, sterols can also be classified as 24-desmethylsterols, 24-methylsterols and 24-ethylsterols. It is also of importance that the introduction of an alkyl group at C24 generates a new chiral centre and thus C-24 epimeric pairs can occur (e.g., campesterol/22-dihydrobrassicasterol, crinosterol/bassicasterol, sitosterol/clionasterol, and stigmasterol/poriferasterol) [43]. In PS, 24α(24*R*)-methyl (e.g., campesterol) or 24α(24*R*)-ethyl (e.g., sitosterol) substituents are the most common (apart from stigmasterol, which has 24β(24*S*) configuration due to its C22=C23), whilst 24β(*S*)-ethyl and 24β(*S*)-methyl are the most common in algae and fungi, respectively [44].

In general, PS are found in their non-esterified forms, but they can have conjugated counterparts. Similarly, to what happens with cholesterol in animal tissues, PS can be esterified with long-chain fatty acids forming fatty acid sterol esters (e.g., steryl palmitate in No Figure 1A). Other PS derivatives include steryl glucosides (SG), formed by β-glycosidic linkage between the hemiacetal carbon of a monosaccharide and the 3′-hydroxyl group of PS (Figure 1B), and acyl steryl glycosides (ASG), where further acylation of the sugar moiety occurs (e.g., steryl glucopalmitate in Figure 1C). Glycosylation is specific to plant, fungi and some yeast species membranes [45], even though a few exceptions were reported e.g., in vertebrates [46] and bacteria [47]. Among sterol conjugates, steryl glucosides (also known as sterolins) are yet to be fully characterized. In fact, SGs of C4-monomethyl sterols have not been reported in higher plants until 2018 [48]. Steryl galactosides, such as β-sitosteryl β-d-cellobioside (3-β-sitosteryl β-d-glucopyranosyl-(1→4)-β-d-glucopyranoside), were also described [49]. Finally, despite being generally less abundant, phenylpropanoid esters of PS, such as ferulates [50], coumarates [51], caffeates [52], cinnamates and sinapates (24-methylenecholesteryl) [53] have also been reported in plants.

### 2.2. Biosynthesis of PS and Derivatives

PS share a common biosynthetic origin with other terpenoids that is linked to the formation of isoprenoids: the five-carbon isoprene-like units that are the very early precursors of PS. At present, there is a consensus on two possible pathways for isoprenoid formation: via the plastidial 2-*C*-methyl-d-erythritol-4-phosphate (MEP) pathway or through the cytosolic (and probably also peroxisomal) mevalonate (MVA). The latter can be simplistically summarized in three main steps: (1) the condensation of three molecules of acetyl-CoA form 3-hydroxy-3-methylglutaryl-CoA (HMG-CoA) catalysed by acetoacetyl-CoA thiolase (AACT) and HMG-CoA synthase (HMGS), (2) HMG-CoA reduction to MVA by HMG-CoA reductase, and (3) a double phosphorylation with a subsequent decarboxylation that leads to isopentenyl diphosphate (IPP) formation (isomerism of IPP to dimethylallylpyrophosphate (DMAPP) via IPP isomerase, to balance the pool sizes of these intermediates, is also possible). As for the plant MEP pathway, the key control steps can be summed up to condensation of pyruvate with glyceraldehyde 3-phosphate (GAP) to produce 1-deoxy-d-xylulose-5-P (DXP), reduction of this product to MEP and the production of isopentenyl diphosphate (IPP) and its isomer, dimethylallylpyrophosphate (DMAPP), catalysed by 4-hydroxy-3-methylbut-2-enyl diphosphate reductase. HMG-CoA reductase and DXP synthase are considered the rate-limiting enzymes in the MVA and MEP pathways, respectively [44,54].

In a further step of PS formation three C5 IPP units join head to tail leading to a C15 compound, farnesyl pyrophosphate (FPP), and thus ending the known “pre-squalene” stage. Two farnesyl pyrophosphates are then joint to form squalene. The C-30 olefin is then converted to squalene 2,3-epoxide, that undergoes cyclization into 24-desalkyl sterols, key precursors that suffer further modifications and receive the extra C24-methyl or ethyl groups by the action of different sterol methyl transferases (SMTs), which are considered to be rate-limiting enzymes in PS (post-squalene) pathway. In plant, the role of the key precursor is generally played by cycloartenol while in animals the synthesis is made with lanosterol as the intermediary. However there has also been reported direct evidence of PS formation via lanosterol in plants (1.5% contribution in *Arabidopsis*), even though not under normal conditions [55].

**Figure 2:**
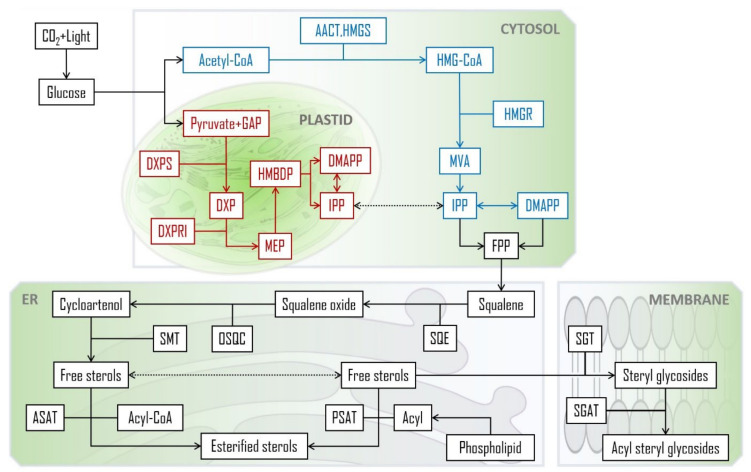
PS biosynthetic pathways leading to the main PS species in plants [44,54,56,57]. CoA: coenzyme A; AACT: acetoacetyl-CoA thiolase; HMG-CoA: 3-hydroxy-3-methylglutaryl-CoA; HMGS: HMG-CoA synthase; GAP: D-glyceraldehyde 3-phosphate; DXP: 1-deoxy-d-xylulose-5-P; DXPS: DXP synthase; DXPRI: reductoisomerase; HMGR: HMG-CoA reductase; MVA: mevalonate; MEP: 2-*C*-methyl-d-erythritol-4-phosphate; HMBDP: (*E*)-4-hydroxy-3-methylbut-2-en-1-yl diphosphate; DMAPP: dimethylallylpyrophosphate; IPP: isopentenyl diphosphate; FPP: farnesyl pyrophosphate; SQE: squalene epoxidase; OSQC: oxidosqualene cyclase; SMT: sterol methyl transferase; ASAT: acyl-CoA:sterol O-acyltransferases; PSAT: phospholipid:sterol O-acyltransferase; SGT: sterol glycosyltransferases; SGAT: steryl glucoside acyltransferase; ER: endoplasmic reticulum.

PS can also be esterified with fatty acids via the dominant route catalysed by acyl-CoA: sterol O-acyltransferases (ASATs), where acyl-CoAs are used as fatty acid donors, or via an alternative path with acyl groups derived from phospholipids, by means of a phospholipid: sterol O-acyltransferase (PSAT) enzyme [56,57]. SE’s fatty acyl chains can range from C12 to C22, but palmitic, stearic, oleic, linoleic and linolenic acids are the most common [58]. The already mentioned glycosylation of free PS and acylation of SGs take place mostly at the cellular membrane, catalysed by sterol glycosyltransferases (SGT) and steryl glucoside acyltransferases (SGAT), respectively. A summarized scheme of the main biosynthetic steps and their general cellular compartmentalization is depicted in Figure 2. The specific enzymes, intermediary modification steps and respective structures can be found elsewhere [59,60].

### 2.3. Functions of Sterols in Plants

The 24-alkyl PS are structural components of cellular membranes, where they interact with phospholipids and proteins, participating in the regulation of membrane fluidity, permeability, and signalling [61]. They are also present in specific membrane regions, the lipids rafts, where stigmasterol and especially sitosterol seem to have important roles, such as regulation of membrane adaptation to thermal shocks [62]. Another example of PS′s function at the membrane level can be seen in the modulation of ATPase activity (e.g., membrane of maize roots), where the mechanism resembles the interaction of cholesterol with animal Na^+^/K^+^-ATPase [63]. Additionally, PS are involved in the biosynthesis of specialized metabolites e.g., brassinosteroid synthesis, where campesterol was shown to be a precursor in many plant species [64] and plant cholesterol as the precursor for steroidal glycoalkaloids (SGAs) and phytoecdysteroids [65]. Also, C28- and C29-PS metabolize to the plant defence-related withanolides (from 24(28)-methylene cholesterol) [59]. Studies on *Arabidopsis* mutants have made a remarkable contribution to the present knowledge of PS function at cellular and subcellular level. To name a few, proper sterol profile importance has been shown in embryonic pattern formation [66], cell division [67], cell elongation [68], cell polarity [69], cellulose accumulation [70,71], regulation of oleosome biogenesis [72], and interaction between sterol biosynthesis and ethylene signalling [73,74]. PS are also involved in responses to biotic [75,76,77] and abiotic stresses, like drought [78,79,80], metal tolerance [81], UV radiation [82] and thermal shocks [83,84,85,86,87]. Finally, steryl esters are mostly linked to storage and transport roles [57,58].

### 2.4. Oxysterols

**Figure 3:**
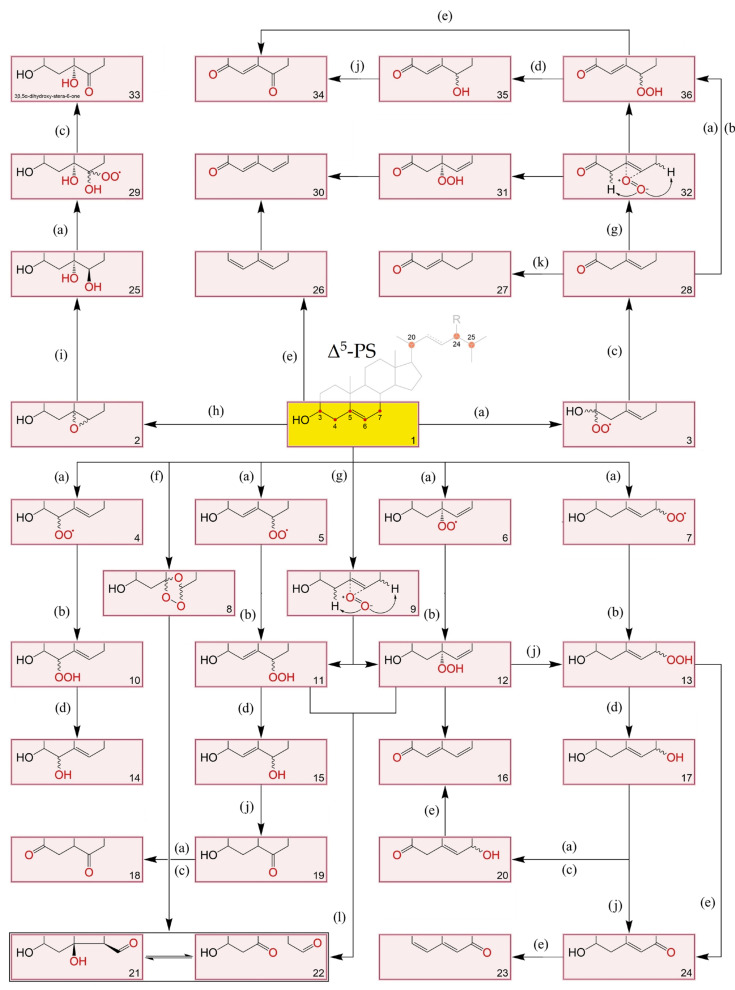
A, B-ring transformations during different sequential reactions in oxidation of Δ^5^-sterols [90,92,93,109,110]. (a): +O_2_, -HO_2_^•^, +O_2_; (b): +RH, -R^•^; (c): +RH, -R^•^, -H_2_O_2_; (d): -HO^•^, +RH, -R^•^; (e): -H_2_O; (f): +O_3_; (g): +^1^O_2_; (h): +ROO^•^, -RO^•^; (i): +H_2_O; (j): rearrangement; (k): isomerization & conjugation; (l): Hock-cleavage mechanism. 1: Original Δ^5^-PS structure where R-H: cholesterol; R-CH_3_: campesterol; R-CH_3_ with C22=C23: brassicasterol; R-CH_2_CH_3_: sitosterol; and R-CH_2_CH_3_ with C22=C23: stigmasterol; 2: 5α,5α- and 5β,5β- epoxy-sterols; 3: Intermediary hydroperoxyl radical; 4, 5, 6 and 7: Intermediary hydroperoxyl radicals at C4, C6, C5 and C7 positions, respectively; 8: sterol ozonides: 5,7α-epidioxi-5-α-B-homo-6- and 5,7β-epidioxi-5-β-B-homo-6- stanols; 9: perepoxide transition state formed via singlet oxygen (^1^O_2_) ene-addition to C5=C6; 10: 3β-hydroxystera-5-en-4α- and 3β-hydroxystera-5-en-4β- hydroperoxides; 11: 3β-hydroxystera-4-en-6α- and 3β-hydroxystera-4-en-6β- hydroperoxides; 12: 3β-hydroxystera-6-en-5α-hydroperoxide; 13: 3β-hydroxystera-5-en-7α- and 3β-hydroxystera-5-en-7β- hydroperoxides; 14: stera-5-en-3β,4α- and stera-5-en-3β,4β- diols; 15: stera-4-en-3β,6α- and stera-4-en-3β,6β- diols; 16: stera-6-en-3β,5α-diol; 17: stera-5-en-3β,7α- and stera-5-en-3β,7β- diols; 18: stera-3,6-dione; 19: 3β-hydroxystera-6-one; 20: 7α- and 7β- hydroxystera-5-en-3-ones; 21: 3β-hydroxy-5-oxo-5,6-secostan-6-al (secosterol A); 22: 3β-hydroxy-5β-hydroxy-B-norcholestane-6β-carboxyaldehyde (secostanol B); 23: stera-3,5-diene-7-one; 24: 3β-hydroxystera-5-en-7-one; 25: stera-3β, 5α, 6β-triol; 26: stera-3,5-diene; 27: stera-4-en-3-one; 28: stera-5-en-3-one; 29: intermediary hydroperoxyl radical; 30: stera-4,6-diene-3-one; 31: 3-oxo-stera-6-en-5α-hydroperoxide; 32: same as 9,3-oxo intermediate; 33: 3β,5α-dihydroxy-stera-6-one; 34: stera-4-en-3,6-dione; 35: 6α- and 6β- hydroxystera-4-en-3-one; 36: 3-oxo-stera-4-en-6α- and 3-oxo-stera-4-en-6β- hydroperoxides.

Oxidation products of both cholesterol and PS have been gaining relevance in clinical data and food quality control [88,89]. As such, it is paramount to consider their structures and formation. The topic has been extensively reviewed but the information on phytosterol oxidation products (POP) is still scarce when compared to data on cholesterol oxidation products (COP) [90,91,92,93]. Nevertheless, it is postulated that like COP, POP can be formed by non-enzymatic or enzymatically catalysed reactions. It seems that the latter has a minor contribution, especially in endogenous formation, since the extra ethyl or methyl group at C24 (or the double bound at the C22) in PS can lead to steric hindrances when compared to cholesterol, making them poor substrates for cellular enzymatic systems. Thus, PS non-enzymatic oxidation by physical processes (such as heating and radiation) and chemical processes (involving reactive oxygen and free radical species) is the main route of the formation of POP [90,91]. Oxysterol (OS) structures and possible A, B-ring oxidation pathways can be found in Figure 3. Elicited from data on oxidation of cholesterol, the main process involves a free radical chain reaction, initiated by hydrogen abstraction from allylic C4 and/or C7, being the last one the most predominant. The formed radicals react with oxygen (^3^O_2_) to produce corresponding peroxyl radicals, which in turn yield the more stable α/β-hydroperoxides (via hydrogen atom donors such as unsaturated lipids). The latter can attack the Δ^5^ double bond leading to secondary oxidation products such as epimeric epoxides that in turn convert to triols via epoxy ring opening by hydration. In accordance the main reported POP are 7-keto, 7-hydroxy and 5,6-epoxy epimers, but derivatives of PS triols have also been identified in some works [94,95]. Given the already mentioned mechanisms, saturated sterols are thus less susceptible to oxidation. Nonetheless stanol oxides have also been characterized in purified rapeseed oil and tripalmitin matrices, including epimers of 7- and 15-hydroxysitostanol and 6- and 7-ketositostanol [96]. O–H and C–H bond dissociation enthalpies (BDE) reveal that theoretically Δ^7^-sterols are more susceptible to oxidation than Δ^5^-sterols and confirm that the dominant sites of oxidation attack are the C7–H bond in Δ^5^-sterols and the C14–H in Δ^7^-sterols [97]. Other oxidation reactions, initiated by highly reactive oxygen species such as singlet oxygen (^1^O_2_) and ozone (O_3_) have also been recognized in sterols [93]. However, the major aldehyde product of sitosterol ozonization has been only recently characterized with a structure similar to secosterol aldehydes previously described for cholesterol [98]. In addition, tertiary carbons in the side chain can also be prone to attacks, including C20 and C25 in the cholesterol molecule, and C24 in major PS. Reports on side-chain oxidation products of PS are scarce but 24- and 25-hydroxy-PS have been well documented in thermo-oxidation of sitosterol, campesterol [99] and stigmasterol [100]. Also, diepoxydes (5,6:22,23) of both brassicasterol and stigmasterol were found in an experiment which also confirmed that unsaturated lipids are readily epoxidated in contact with hydroperoxide triglycerides (TGs), in the absence of molecular oxygen [101].

On the latter note, it is crucial to consider that several factors can influence the formation of sterol oxidation products (SOP), including heating temperature and time, processing and storage, type of PS structure and esterification, food matrix saturation degree, water presence, exposure to ionizing radiation or natural/artificial light (can be related to storage) and oxidizing agents used in food processing (oxidizing agents per se such as hydrogen peroxide in egg or cheese processing, or pro-oxidizing food additives such as metal ions, pigments, or enzymes) [92,102,103,104,105,106]. The PS esters in fortified foods can be even more prone to oxidation than their free counterparts, depending on the fatty acid moiety [107], even though a protective effect can be observed on the sterol moiety [108]. In any case, both inter, and intramolecular oxidation mechanisms should be considered in fatty acid steryl esters [93].

### 2.5. Disteryl Ethers

**Figure 4:**
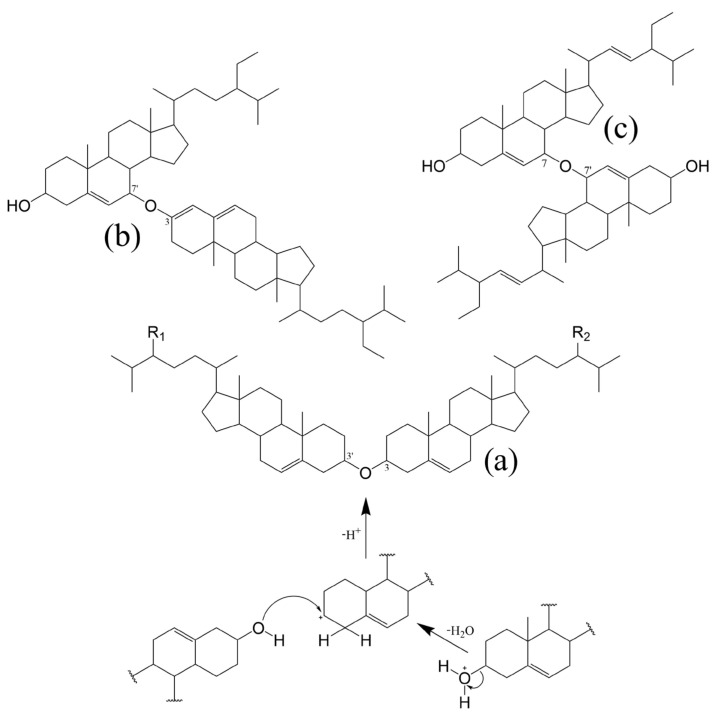
Examples of 3β,3′β-disteryl ethers (DE) and DE formed with POP subunits; proposed mechanism for acid-catalysed dehydration of sterols during bleaching, leading to the formation of 3,3′-DEs. (**a**) DE with same PS subunits: R_1_, R_2_-H, dicholesteryl ether; R_1_, R_2_-CH_3_, dicampesteryl ether; R_1_, R_2_-CH_2_CH_3_, disitosteryl ether. DEs with mixed PS subunits: R_1_-H, R_2_-CH_3_, 3,3′-campesterylcholesteryl ether; R_1_-H, R_2_-CH_2_CH_3_, 3,3′-sitosterylcholesteryl ether; R_1_-CH_3_, R_2_-CH_2_CH_3_, 3,3′-sitosterylcampesteryl ether. (**b**) 3,7′-sitosta-3,5-dienylsitosta-3′β-ol ether proposed by Rudzinska et al. (2010) [112]; (**c**) 7,7′-distigmasta-3,3′-diol ether hypothesised by Struijs et al. (2010) [113].

Other interesting structures that were reported to be formed by bleaching of commercial oil and table margarine, are the 3β,3′β-disteryl ethers (DE) [111]. Acid-catalysed formation of DE during bleaching involves an initial formation of a charge transfer complex at 3β-hydroxyl of the sterol followed by dehydration and finalized by a nucleophilic substitution to form the corresponding 3β,3′β sterol dimer (Figure 4) [110].

Recent research corroborates that such dimers can indeed be produced by high-heating (highest content at 220 °C for 1 h) of sterol-rich samples, being the 3β-hydroxy group and the C5–C6 double bond in one of the sterol molecules the required conditions to be met [114]. In a following work, the authors present data on oxidized 3β,3′β-disteryl ethers formed by heating, when catalysed by metal ions. Interestingly the only dimer found in plant-origin samples (rapeseed oil) was the 7-ketositosteryl-sitosterol ether [115]. But the idea of dominant monomeric oxidation products being one or both subunits is not new since the formation of PS dimers through ether 3,7′ [112] and 7,7′ – linkages [113] was already proposed in earlier publications. Examples of DE can be found in Figure 4. Finally, it should be mentioned that oligomer accumulation was also registered throughout prolonged oxidation of fatty acid steryl esters, confirming the significance of polymerization reactions in oxidation of steryl esters as well [116].

### 2.6. Synthetically Modified PS

**Figure 5:**
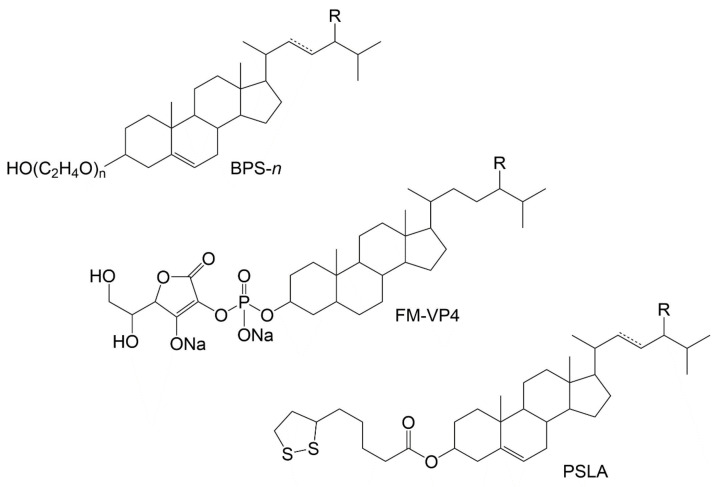
Examples of modified PS. BPS-*n*: PS surfactants commercialized by Nikkol Group where *n* represents the degree of ethoxylation that will determine the properties. While lower numbers (e.g., BPS-5) provide emollient properties, higher degree of ethoxylation (e.g., BPS-30) is needed for solubilising capabilities. The products can have different % of PS such as campesterol (R-CH_3_), sitosterol (R-CH_2_CH_3_) and stigmasterol (R-CH_2_CH_3_ and C22=C23) [120]. FM-VP4: disodium ascorbyl phytostanyl phosphates, with campestanol (R-CH_3_) and sitostanol (R-CH_2_CH_3_) as the possible PS moieties; PSLA: conjugates of PS and lipoic acid synthesised and characterized by Madawala et al. (2012) [122]; R-CH_3_: campestanyl lipoate; R-CH_2_CH_3_: sitostanyl lipoate, R-CH_3_ with C22=C23: brassicasteryl lipoate, and R-CH_2_CH_3_ with C22=C23: stigmastanyl lipoate.

Many synthetic PS and stanol conjugates have been developed to bolster PS commercial suitability via higher melting temperature, lower crystallization temperature, better thermal stability, enhanced water-solubility, improved emulsifying activity and stability, increased bioavailability, or antioxidant activity [117]. While altering PS original physical and/or chemical properties is an expected outcome, the main goal seems to be linked to paradigmatic PS uses, being hydrophilic phytostanol analogues such as disodium ascorbyl phytostanol phosphates (FM-VP4 in Figure 5) [118] or phytosteryl succinyl sucrose esters [119], good examples. PS have also been used as hydrophobic tails in sterol-based surfactants known as sterol ethoxylates. Commercialized by Nikkol, Japan (for further information see BPS-*n*, in Figure 5), they are primarily used in cosmetic preparations [120,121].

However, most reported PS’s synthetic modifications are based on mimicking structures encountered in natural sources, including dozens of studies on PS esterification products with different fatty acids and phenolic acid moieties, already reviewed by other authors [117]. Enzymatic/nonenzymatic esterification or transesterification with fatty acids is a classic example since it can improve fat solubility, enabling an easier incorporation into the food matrix, especially with the introduction of a longer fatty acid chain and a higher number of double bonds. On the other hand, phenolic acid esters can be used to counter PS’s lipophilic nature, which results in minimal solubility in aqueous media, and provide antioxidant activities [123]. The latter category includes the synthetic counterparts of above-mentioned natural structures (e.g., ferulate [124], sinapate [125], cinnamates [126,127] and caffeate [128]) and other phenolic moieties like galloylate [129] vanillate and 4-hydroxybenzoate [123]. Reports on conjugates with endogenous-like antioxidants such as lipoic acid and its dithiol form, dihydrolipoic acid, were also made [122,130].

## 3. Dietary Sources and Daily Intake

The bulk of dietary PS intake comes from vegetables oils, margarines and milling products, but a typical Western diet includes other sources such as vegetables, fruits, and nuts, amounting to PS intakes in the range between 200 and 300 mg/day and in vegetarians it can go up to 500–1000 mg/day [131]. In either case, to achieve the recommended intake of 2 g of PS for a hypocholesterolemic effect, PS supplementation is required. The most abundant PS species in most edible sources are the desmethyl Δ^5^-sterols i.e., sitosterol, campesterol and stigmasterol with their conjugates, SG (e.g., cereals rich in SGs), and in lesser degree their stanol counterparts [132]. The richest edible sources of sitosterol are mostly vegetable oils such as olive (111–147 mg/100 g), peanut (150–223 mg/100 g), sesame (361–374 mg/100 g) and soybean (213–229 mg/100 g) oils [133]. Among edible foods, some of the richest reported sources of sitosterol are avocado (62-98 mg/100 g) [134] and pistachio nuts (107-126 mg/100 g) [135]. Some foods have specific sterol species e.g., spinasterol and Δ^5^-avenasterol in spinach (*Spinacia oleracea*) and oats (*Avena* L.), respectively. Brassicasterol (from genus *Brassica* in e.g., rapeseed oil) is also to be considered in certain oils and their quality assessment [136]. Finally, while cholesterol is usually a minor constituent of the sterol fraction in plants, it can be substantial in certain families e.g., *Solanaceae* (potato and tomato as the main edible examples) [137]. Total PS content of common foods in a non-vegetarian diet, compared with the estimated daily intake and recommended intake for hypercholesterolaemia control, can be found in Table 1.

Oxysterol content in food, including COP and POP, varies with the diet. Considering mixed diets, COP should be around 1% of total cholesterol [91,138] and POP between 0.5–2.3% of total PS as reported by EFSA (European Food Safety Authority) [139] based on oxidation rates of cooking experiments with PS products. However, several works report much lower POP estimations, at around 0.1–1.0% [91,138,140,141]. Thus, even in the case of PS supplementation, the total daily POP intake, established on recommended intake for the hypocholesterolemic effect (2 g), is between 2.0–45.6 mg/day.

## 4. Absorption and Metabolism of PS and Derivatives

The absorption of deuterium labelled sitosterol and campesterol, as well as their respective stanols (sitostanol and campestanol) were reported to be minimal: 0.512 ± 0.038%, 1.89 ± 0.27%, 0.0441 ± 0.004% and 0.155 ± 0.017%, respectively, based on studies using isotopically labelled PS [142]. Even though the real values can be higher (transfer of radioactivity discussed in Kritchevsky et al. (1965) [143]) the results are corroborated by the oral bioavailability of sitosterol (0.41%) determined by Duchateau et al. (2012) [144], and the average baseline plasma concentrations (nearly 300-fold lower than cholesterol: 0.288 mg/dL for sitosterol and 0.524 mg/dL for campesterol) reviewed in a meta-analysis by Ras et al. (2013) [131]. These values fall within the reference intervals for Japanese healthy subjects reported by Yoshida et al. (2020), being higher in women than men: 0.099–0.388 and 0.214–0.743 mg/dL in men, and 0.103–0.445 and 0.219–0.834 mg/dL in woman, for sitosterol and campesterol, respectively [145]. Plasma PS concentrations in elderly subjects were reported to have a general tendency to be higher (e.g., 0.330 ± 0.170 mg/dL and 0.320 ± 0.170 mg/dL for sitosterol concentrations in females and males, respectively), even though the picture can change depending on the clinical history and/or prior PS supplementation [146]. There is a lack of information on infants, but PS were found to rapidly accumulate in neonates (1.68 mg/dL for sitosterol within 1.5 days) [147]. Oxidized phytosterols (OPS) seem to have even lower concentrations, in the range between 7.0 and 452.0 ng/dL, according to several cohesive studies [148,149,150], being 7-oxositosterol structures the most predominant. This results in concentrations of up to 10 times lower than COP [151]. OPS are not preferentially transported into tissues according to human and animal models [152] and based on recent data their absorption may even be lower since the results imply that OPS plasma concentrations primarily result from hepatic spill-over into the circulation [153].

**Figure 6:**
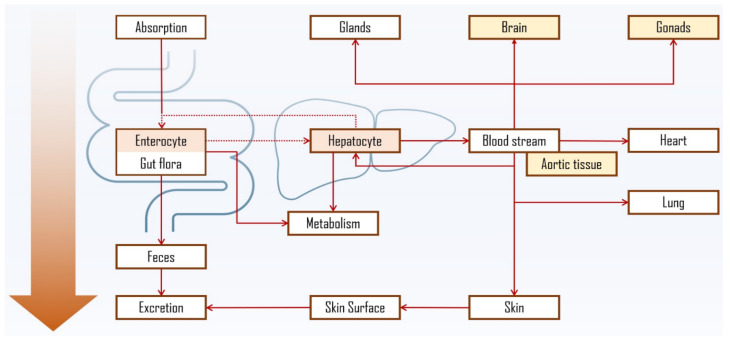
Proposed PS distribution and accumulation via oral ingestion [161,162,163,165,166]. Dashed lines represent the entero-hepatic interaction, further discussed in Figure 7. Tissues and organs, reported to have the most PS accumulation, are marked in colour, being the highest in enterocyte and hepatocyte.

In fact, PS liver metabolism and enzymatic oxidation were confirmed in earlier studies [154,155] with bile acids found in human plasma and bile after intravenous injection of labelled sitosterol, which converted into the same primary structures as cholesterol, i.e., cholic and chenodeoxycholic acid [156]; non-conventional bile acids (e.g., acidic metabolites) were also observed [142,156,157]. Most of the sitosterol was found to be rapidly secreted into the bile in its free form, thus leading to mainly faecal excretion, which was also confirmed for other common PS [158]. PS biliary secretion rate in male volunteers was shown to support the inverse relationship between hepatic clearance and intestinal absorption, with sitosterol’s being quicker (1.23 mg/h) when compared to campesterol (0.76 mg/h) [159]. It is worth mentioning that in situ biohydrogenation of sterols to stanols and/or stanones in human digestive tract, analogous to well-known transformation of cholesterol to coprostanol, should also be considered [160]. In fact, PS and their corresponding stanols were found in faecal samples of healthy individuals [161]. Figure 6 depicts the proposed PS distribution via oral ingestion, including PS accumulation in intestinal epithelia, liver, aortic tissue, adrenal glands, brain and skin [162,163]. The latter has also been proposed not only as another route for PS absorption [164] and elimination [165], but also as OPS formation site in healthy subjects via oxidation catalysed by UV light of PS absorbed from cosmetic products.

Research data also suggests PS’s accumulation in brain tissue [166,167]. However, the exact processes of PS metabolism and tissue distribution, as well as its influence on cholesterol homeostasis, are not fully elucidated. Nevertheless, entero-hepatic interaction, similarly to cholesterol, has a central role. In fact, the absorption of dietary cholesterol and PS is regulated by shared mechanisms at hepatocyte and enterocyte levels, where ATP-binding cassette (ABC) protein transporters have a central role [168]. While low PS plasma concentrations in healthy individuals are primarily a result of normal intestinal ABCG5/G8 action, autosomal recessive mutations in ABCG5/G8 lead to rare phenotypes in individuals characterized by excess concentrations of circulatory PS, a condition known as sitosterolemia [169].

**Figure 7:**
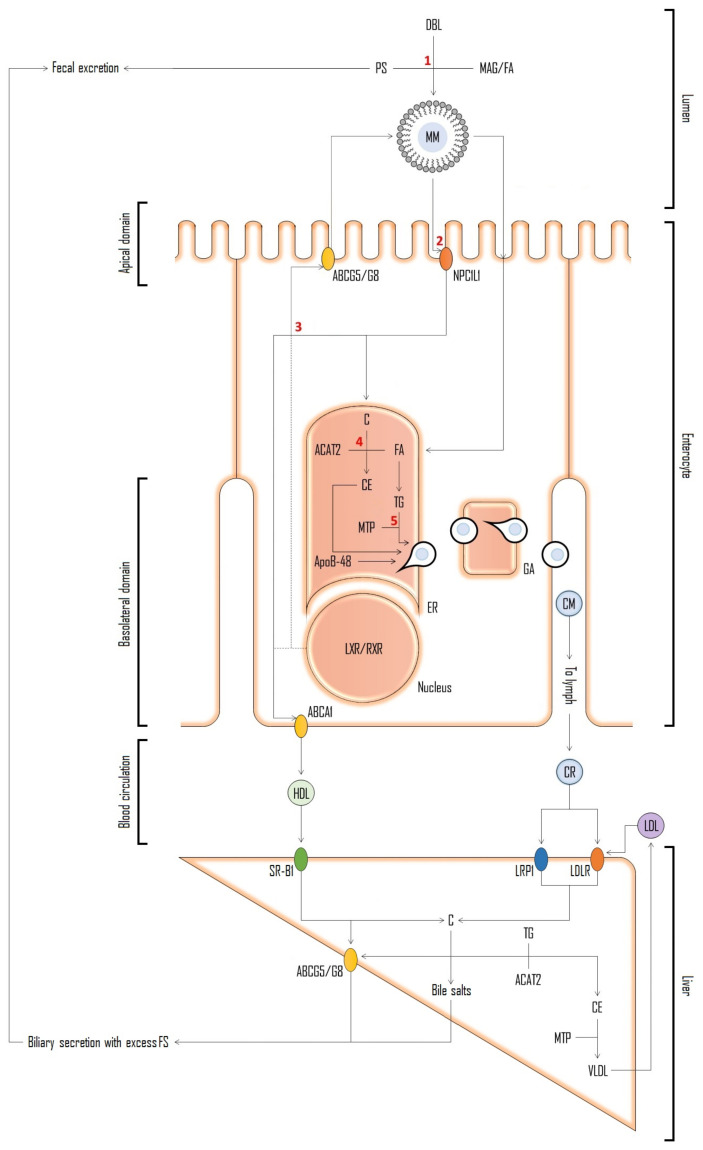
Schematic representation of resumed entero-hepatic mechanisms shared between cholesterol and PS [175,176,177]. Abbreviations: free cholesterol (C); fatty acids (FA); monoacylglycerols (MAG); mixed micelles (MM); Niemann-Pick C1 Like 1 protein (NPC1L1); endoplasmic reticulum (ER); cholesteryl esters (CE); acyl coenzyme A: cholesterol acyltransferase-2 (ACAT2); chylomicrons (CM); triglycerides (TG); microsomal triglyceride-transfer protein (MTP); ATP-binding cassette protein (ABC) transporters (ABCG5/G8); high-density lipoproteins (HDL); nuclear liver-X receptor/retinoid-X receptor (LXR/RXR); low-density lipoprotein (LDL); scavenger receptor class B member 1 (SR-B1); low-density lipoprotein receptors (LDLR); LDLR related protein 1 receptors (LRP1); very low-density lipoprotein (VLDL). Red numbers refer to possible interference of PS on cholesterol pathway and the basis on possible explanations for PS hypocholesterolemic action proposed over the years [177]. 1: competition for incorporation into mixed micelles; 2: competitive inhibition of cholesterol uptake by brush boarder membrane affecting NPC1L1 uptake; 3: activation of LXR resulting in up-regulation of cholesterol efflux transporter genes; 4: inhibition of cholesterol esterification via ACAT2; and 5: interference with cholesterol incorporation into nascent CMs.

The fact that PS share the same intestinal and hepatic transporters with cholesterol, but are not synthesized in the human body, makes them a much affordable option as markers for assessment of cholesterol absorption [170]. The mentioned mechanisms are initiated in the intestine lumen (Figure 7). Dietary and biliary lipids, including enzymatically digested free cholesterol, fatty acids and monoacylglycerols, are incorporated into mixed micelles (MM) which enter the enterocyte apical domain. At this point Niemann-Pick C1 Like 1 (NPC1L1) protein plays a major role in the transport of sterol compounds to the cytoplasm and endoplasmic reticulum (ER). In ER, cholesterol is re-esterified into cholesteryl esters (CE) with the help of Acyl coenzyme A: cholesterol acyltransferase-2 (ACAT2) and, together with triglycerides (TG), is incorporated into apoB48 containing chylomicrons (CM) by microsomal triglyceride-transfer protein (MTP). CMs are secreted into lacteals at the basolateral enterocyte domain and enter the venous circulation. PS follow the same path being absorbed into enterocytes in micelles, however being poor substrates for esterification by endoplasmic ACAT2 [171,172] most of the absorbed PS are secreted back to the intestinal lumen by ABCG5/G8 protein transporters [173]. The remaining PS can be integrated into CM as described and enter circulation or be incorporated in their free form along with cholesterol into high-density lipoproteins (HDL). PS secretion into nascent HDL carriers is supported by the observed enhancement of basolateral efflux of β-sitosterol upon nuclear liver-X receptor/retinoid-X receptor (LXR/RXR) activation in CaCo-2 cell line [174]. In fact, the highest concentrations were found in HDL and low-density lipoprotein (LDL). Cholesterol uptake into hepatocytes occurs mostly from HDL via scavenger receptor class B member 1 (SR-B1), and from CMs via low-density lipoprotein receptors (LDLR) and LDLR related protein 1 (LRP1) receptors. In hepatocyte, cholesterol is esterified and incorporated into very low-density lipoprotein (VLDL) that can be delivered to peripheral tissues [175].

## 5. Toxicity and Safety Assessment of PS Products

PS, PS stanols and their esters (PSE) have approved health claims by both the European Commission (EC) and FDA with the latter classifying PS as GRAS (Generally Recognized as Safe) [13]. Additionally, PS products have been evaluated by scientific authorities such as Joint FAO/WHO Expert Committee on Food Additives (JECFA) [178], European Food Safety Authority (EFSA) [139] and the Scientific Committee on Food (SCF) [179]. From the presented toxicological data, the conclusion is that PS pose no risk, even though the bulk of the presented studies were focused on selected high-purity PS products. Aside from the obvious contraindications in sitosterolemia conditions, in general, the main long-term safety issues are concerned with PS interference with the absorption of carotenoids and the formation of POP. Reduction of plasma carotenoid levels while under PS supplementation can easily be bypassed by increasing the consumption of fruits and vegetables [180]. As for oxidation products, growing concerns on a possible relationship between POP and cytotoxicity have been reported [88,89,91,139,152,181,182,183]. The concern is evident in the last EFSA report on the safety of extension of PSE use, where the stated PS oxidation rates for foods in cooking experiments (0.5–2.8%) were used for the estimation of the daily safe level of 0.64 mg POP/kg of body weight [139]. The result can be even more impactful considering the maximum PS recommended intake of 3 g (which can result in POP values exceeding the established daily safety level), thus requiring more long-term randomised, controlled clinical trial data to establish the safety of the intended extension use of PS under the proposed requirements. Additionally, it remains an open question if safety evaluations mostly focused on PSE or undefined mixtures of PS can be misleading, especially when assessing a single PS component e.g., the evaluation of stigmasterol-rich additive using PSE-based studies [184], while the cytotoxicity for the heated free stigmasterol was reported being higher than that for the respective heated sterol esters [108]. Finally, since the bulk of the population has a mixed diet, the full picture of the safety evaluation cannot be made without including COP-related variables, especially if considering the many oxidation propagation reactions discussed above.

## 6. Analysis of PS and Conjugates

### 6.1. Analytical Approaches for PS Analysis

Identification of PS requires a multiple approach including sample pre-treatment, extraction and analytical procedures. The sample nature, and target PS specie to be identified and quantified are the most important considerations to select the analytical methodology. PS content is another aspect to consider since it is directly connected to quantification limits and needed sample amount.

#### 6.1.1. Sample Pre-Treatment

Pre-treatments can be used prior to extraction, such as acidic hydrolysis or saponification, either to release total PS or to enhance the efficiency of extraction. In samples with complex polysaccharide–protein matrices (found in cereal or wet vegetable tissues) a first step of acidic hydrolysis can be used to cleave the glycosidic linkages, such as in SGs, or the ester bond in esterified sterols, and thus allowing the release of PS, that then will be extracted using nonpolar solvents. This pretreatment approach is good for the identification of total PS, but not for PS conjugates. However, acidic hydrolysis may have some drawbacks. In samples dominated by PS susceptible to acid-catalyzed isomerization (e.g., some nuts and seeds) [185], such as Δ^7^-sterols, enzymatic hydrolysis should be considered instead, providing that target PS-containing extracts or SG/ASG fractions can be efficiently obtained from the matrix [186].

Alkaline hydrolysis is mostly used to release the PS from a sample rich in fat, oil, or lipid extract samples, where original PS species mainly exist as free PS or as phytosteryl esters (PSE) [187]. Saponification also cleaves the triacylglycerides (TAG) and releases the fatty acids, being the released free PS separated from the fatty acids by liquid-liquid extraction. This step is very important particularly considering that PS are usually a minor constituent, comprising <1% of the matrix but they can be several folds higher in PS-fortified foods (e.g., fortified fat spreads with ca. 8% PS) [139] or very low in biological samples such as blood serum [149].

After saponification, the unsaponifiable material that includes the free sterols, is extracted by liquid extraction with one or more nonpolar solvents such as hexane, n-heptane, chloroform, or diethyl ether. The extraction can be repeated to maximize the yield. Saponification is thus essential to remove TAGs, enhance the extraction of PS and can be much faster than standard liquid extraction in solid samples where PS can be strongly bound or even captured in solid matrices and/or particles [188].

Relatively harsh saponification treatments can result in degradation of some labile sterols such as dienes [189] and for sterol oxide determination a gentle cold saponification is normally advised to avoid artifact formation [190,191]. Saponification can also allow an easy release of PS from conjugates in natural extracts and biological samples for total PS analysis; the process can be easily accelerated using sonication, resulting in drastically reduced analysis time [192]. However, ultrasound should be used with caution since it is accompanied by complex, nonlinear phenomena [193], and a change in the distribution of products or even the formation of new species cannot be entirely overruled [194,195]. But saponification, as well as acid hydrolysis, promote the release of sterols, being suitable methods for the profiling of total sterols, however lacking information on the structure of PS conjugates.

#### 6.1.2. Extraction of PS and Derivatives

As already discussed, after the saponification step the unsaponifiable matter is extracted with one or more nonpolar solvents. However, direct extraction from the sample, without acid or alkaline pre-treatments, can be the first treatment step to obtain an extract with the sterols, which has the advantage of extracting the free sterols as well as the conjugate forms of the PS. The most widely used extraction methods for PS from vegetal matrices are the classic Soxhlet (with n-hexane, petroleum ether and ethanol as the most common solvents), and the well-established solvent extraction methods such as Folch [196] and Bligh and Dyer [197]. Alternatively, more environment friendly approaches that avoid chlorinated solvents are available, including methods based on hexane: isopropanol systems, successfully used for PS and OPS determination in vegetables [141], and other more sustainable approaches such as supercritical fluid extraction (e.g., supercritical CO_2_) [198].

Independently of the protocol or target PS analytes, there is always a need to prevent PS losses, conversions, and artifact formation. The latter aspect is especially important in OPS determinations, where temperature, light, and oxygen should be controlled during the whole protocol, including extraction, to avoid oxidation processes already discussed above. Thus, antioxidants can be added to the extraction solvents, such as butylhydroxytoluene (BHT) used from 0.05% to 1% [148]. Similarly, in PS ferulate determinations some precautions concerning direct light can also be beneficial since extraction and isolation in “white” fluorescent light can prevent isomerization of ferulates [141,199].

#### 6.1.3. Fractionation for Enrichment and Isolation of PS and Conjugates

In most cases, the lipid extract obtained from hydrolysis/saponification or lipid extraction have the sterol species mixed with other lipophilic compounds and in some cases even non-lipid constituents. In fact, the “unsaponifiable” fraction obtained after saponification procedures can contain in addition to sterols, also free fatty acids, triterpenes, carotenoids and tocopherols. Even though derivatization without further clean-up/pre-separation can be done in a gas chromatography (GC) analysis for total lipophilic profiling without much interference of the mentioned compounds, in extracts obtained from samples rich in lipophilic species, such as crude oils, it may be necessary to purify the sterol fraction from polar unsaponifiable lipids. Similarly, in lipid extracts obtained from the Bligh and Dyer or Folch of biological samples, the total lipid extracts normally include not only sterol derivatives, but may also contain polar lipids (e.g., phospholipids, among others), and non-polar species that include not only SE but also monoacylglycerols, diacylglycerols, and TAGs, that can hamper sterol analysis [200]. Thus, in most samples, fractionation and purification of the target PS fraction may be required and it is especially important for the identification of sterol conjugates. Both, column chromatography and thin layer chromatography (TLC), can be used for separation of the PS classes in plant-based matrices. For example, PS fractions of 4,4’-dimethylsterols, 4-methylsterols, and 4-desmethylsterols can be separated by solid-phase extraction (SPE) using neutral alumina by sequential elution with 80:20, 70:30 and 60:40 (*v*/*v*) of hexane/diethyl ether, respectively [201]. The separation of these three PS fractions was also achieved by using silica TLC plate developed twice in hexane/diethyl ether/acetic acid (70:30:1, *v*/*v*) [202,203] or once with chloroform: diethyl ether 9:1 (*v*/*v*) [204]. The total lipid extracts obtained with dichloromethane: methanol 2:1 (*v*/*v*) were fractionated in four PS groups (SG, ASG, FS, and SE) by silica TLC using a mixture of dichloromethane: methanol: water 85:15:0.5 (*v*/*v*/*v*) as eluent [205]. Column chromatography needs time to set up and can be burdensome when running multiple samples but is well suited for larger amounts of lipids (>200 mg), while for smaller lipid samples preparative TLC or SPE are normally a better choice since they are less time-consuming, easier to run and a higher resolution can be achieved with TLC.

SPE (solid-phase extraction) and SPME (solid-phase microextraction) separations are both ideal choices for samples with very low content of PS, with silica-SPE allowing higher recovery of PS classes, compared to traditional TLC [203], when employing successive elutions with hexane: diethyl ether systems, from an initial ratio of 99:1 to a final of 60:40 (*v*/*v*) [206]. The 4,4-dimethyl sterols can be easily separated from other unsaponifiable material with the same solvent system in a 95:5 (*v*/*v*) ratio [202]. Alumina SPE cartridges can be used very effectively for a small-scale separation of SE and FS in vegetable oils by collecting SEs via elution with diethyl ether: hexane 20:80 (*v*/*v*) and FSs with ethanol: hexane: diethyl ether 50:25:25 (*v*/*v*) [207]. SPE is thus successfully used to perform the concentration and purification of sterol fraction from polar unsaponifiable lipids. Other examples include the use of aminopropyl SPE with chloroform: isopropanol 2:1 (*v*/*v*) in serum samples [208]; and silica SPE with hexane: isopropanol 100:1 (*v*/*v*) and C18 SPE with chloroform: methanol 20:1 (*v*/*v*) elution in samples from cereal (after saponification and extraction) [209]. Fractions of free PS, PS fatty acid esters, and PS phenolic acid esters can be obtained via aminopropyl SPE with n-hexane: diethyl ether 98:2, n-hexane/ethyl acetate 5:95 and n-hexane/ethyl acetate 5:95 (*v*/*v*) followed by MTBE, respectively, while interfering TAGs are removed by n-hexane/ethyl acetate 96:4 (*v*/*v*) [210]. Silica SPE was also shown to be useful for simultaneous determination of tocopherols and sterols in a single analytical run [211,212]. For SOP enrichment, aminopropyl [213] or silica SPE [203] can be employed. The latter may also be used to obtain enriched fractions in SEs, FS and SOP from lipid extracts obtained by Bligh and Dyer by respective sequential elution with hexane: isopropanol 100:0.5 (*v*/*v*) and hexane: isopropanol 100:30 (*v*/*v*) [200]. The separation of SOP from biological samples can be achieved by a combination of C18 SPE and a polymeric reversed phase SPE with a derivatization step in between: the first SPE retains less polar species, such as free sterols and SEs, while SOP and other polar species pass through; after the derivatization (GP-reagent), the second SPE separates SOP from un-reacted reagent for a subsequent final elution of absorbed on column derivatized SOP [214]. 

Other SPE with different and less used stationary phases can be employed for specific purposes, such as to reduce matrix interferences e.g., removal of phospholipids from serum samples with SPE zirconia-coated silica cartridges [192]. A Diol SPE-based (medium-polarity adsorbent used to extract polar samples from non-polar solutions) protocol for SG and ASG separation should also be mentioned, which includes successive elution with heptane: isopropanol using 97:3, 92:8 and 85:15 (*v*/*v*) ratios, which results in fractions of “other sterol groups”, ASGs and SGs, respectively [215]. SPME with coating of the fibre with derivatizing agent can offer a rapid screening of PS in a one-step extraction-derivatization protocol. However, the method should be optimized to offer a good balance of PS recovery and derivatization yield if quantitative information is needed. Polyacrylate-coated SPME fibre was shown to be well adapted for sterol extraction and it was found to be resistant to degradation by BSTFA (N,O-Bis(trimethylsilyl) trifluoroacetamide), a common silylation reagent. The advantages over other analytical PS techniques include the simplicity of the method and the possibility of easy automation [216,217].

#### 6.1.4. Derivatization

Derivatization is essential in most sterol analyses and has proved to enhance many aspects of sterol characterization, especially via various mass spectrometry (MS) methods [218,219,220,221,222]. PS are usually derivatized by silylation prior to gas chromatography (GC) analysis [220], where the formation of sterol trimethylsilyl (TMS) ethers permits to increase the volatility of the target PS and to improve the stability of some species such as the Δ^5,7^-steradienes which tend to be thermally labile, reduce the polarity and interactions with chromatographic column active sites and, most importantly, provides additional structural information of molecular and useful fragmentation ions in a MS analysis. Several silylating agents can be used depending on the method and target sterols. The most common available reagents for silylation are N,O-Bis(trimethylsilyl) trifluoroacetamide (BSTFA) and N-Methyl-N-(trimethylsilyl)trifluoroacetamide (MSTFA) with 1% trimethylchlorosilane [220,223]. Being secondary alcohols, PS are readily silylated but even so the reaction rate can be increased by heating, which is usually done at around 60–105 °C for 30 to 15 min [218,220,223], with overnight room temperature approaches also being described [191]. The choice of the internal standard should be carefully considered, e.g., when using betulin, the preparation of silylating reagent is recommended by adding 1-methylimidazole and N-methyl-N-(trimethylsilyl)-heptafluorobutyramide (MSHFBA) in order guarantee that both hydroxyl groups are silylated (otherwise betulin may show two peaks in the chromatogram) [223]. However, for cholestanol (one OH group), ready to use commercially available BSTFA or MSTFA with 1% trimethylchlorosilane is enough.

In the case of the derivatization of some multi-hydroxylated sterols, such as oxysterols, which have hydroxyl groups occupying vicinal positions, alternative protocols for derivatization at ambient temperatures overnight in the dark (common practice in other type of derivatizations e.g., with GP-reagent [214]) may be recommended [191]. It is important to avoid artifact formation in OPS determinations, to bypass possible steric hindrances and guarantee full silylation e.g., depending on silylating agents 5α,6β-dihydroxycholesterol or 5α,6β-dihydroxysitosterol can result in bis- and tris-TMS ether derivatives due to steric hindrance at 5α-hydroxyl group position [191]. Despite being less popular, alkylation [224] and acylation [225] can be successfully applied for similar purposes with the advantage of more stable final products, especially in the former case.

Derivatization of PS has also been proven to be beneficial in terms of ionization (via charge tagging) and/or fragmentation in analyses by MALDI (matrix-assisted laser desorption/ionization)-MS or (Liquid chromatography (LC))-ESI (electrospray ionization)-MS. Such is the case of the formation of the N-methypyridyl sterol ethers, and sulphated and picolinyl sterol esters with best results for MALDI [226]; and dimethylaminobutyrate sterol esters [227], Girard P [214] and Girard T [228] derivatives, N,N-dimethylglycine [161], picolinyl sterol esters [229] and dansylated products [230] for ESI.

Derivatization employing isotope-labelled reagents can give further possibilities, especially when labelled standards for some sterols are difficult to obtain e.g., in the case of many oxysterol species. This approach can result in analytes with differing mass tags and almost identical behaviour (retention time and MS response) that can be used to characterize one sample against the other [231]. Derivatization-made species with isobaric mass tags can also be exploited in a similar way, but the differentiation is made by characteristic fragments via tandem MS (e.g., in LC-MS approaches). The example is given by Crick et al. (2015) where [^13^C_2_]GP and [^13^C^15^N]GP tagged sterols can be differentiated and quantified one against the other via tandem MS monitoring (selected reaction monitoring) of ions corresponding to pyridine loss [M – Py]^+^ [232].

It should be outlined that most established derivatizations are based on modifications of free hydroxyl groups, being the latter one a requirement. This results in a limited application in esterified forms of sterols. A rather scarcely explored exception to the rule is the derivatization employing radical tagging. Thiyl radical-based addition using thioglycolic acid (TGA), via the already discussed above allylic B-ring hydrogen abstraction, was reported to result in charge tagged 7-thioglycolate sterol derivatives (including most common PS), suitable for MS quantification [233]. In their work, Adhikari and Xia (2017) also present post-tagging MS and MS/MS spectrum of cholesterol acetate as an example of possible derivatization without the access of a free OH group and thus open the discussion of possible applicability in conjugated sterol derivatives. In this context derivatization has also shown to be a vital asset even for specific analytical solutions, such as pinpointing the C=C within unsaturated fatty acyls in cholesteryl ester isomers to enable their identification and quantification, which was shown to be possible via 2-acetylpyridine (2-AP) Paternò-Büchi (PB) reaction charge-tagging [234] and ozone-induced dissociation (gas-phase ozonolysis of mass-selected ions) [235].

Despite many advantages, derivatization can have some drawbacks, including time consuming protocols that frequently require additional steps (e.g., a pre-conversion to 3-oxo derivatives of OH-PS for sterol GP-hydrazones), the risk of introducing trace impurities or increase the chances of artefact formation, (thus some sort of post-derivatization filtration/separation can be a requirement to remove excess reagent) and possible incompatibilities with some stationary phases (e.g., Carbowax (polyethylene glycol)-type columns with silylated compounds) [220]. Additionally, derivatized PS can also have very little difference in polarity with TAGs which can coelute in further steps with desired analytes [211].

#### 6.1.5. Internal Standard Addition for GC-MS Analysis of Silylated PS Derivatives

This step is mandatory, both in targeted or untargeted approaches, to guarantee accurate qualitative and quantitative results in PS analysis. Internal standard (IS) addition is often used to check or optimize protocol conditions, such as PS recovery or derivatization rate, or in case of OPS, to monitor artifact formation. Additionally, it offers certain perks in MS methods, e.g., possibility of monitoring the relative retention time (RRT) of the PS vs. IS or in the case of using split injection in a GC analysis, IS can compensate for possible variations of effective injected amount of sample entering the column (drastic variations in IS peak response (area) can point to possible extraction protocol or injection errors). Self-explanatory, in most cases, IS addition is made at the beginning of the protocol, even before extraction/saponification. In specific situations with more complex initial matrices, in doubt of the achievable degree of saponification, mixing in an SE IS in a preliminary run can help to validate the method, guaranteeing a more accurate estimation of total PS. In general sterol determinations of food matrices, the most used IS are 5α-cholestane, 5α-cholestan-3β-ol, 5β-cholestan-3α-ol and betulin. Depending on the context of the analysis, some of them may present some flaws. Cholestane can have two major drawbacks: depending on TLC used it may not elute with other sterols (but for example betulin does), requiring post-run addition (thus with no compensation for any loss of sterols during the extraction of unsaponifiable matter or during further stages that can require re-extraction of the PS), and contrary to other IS it cannot be silylated (e.g., no possibility to monitor potential problems with the silylation reaction if used) [236]. On the other hand, cholestane elutes early, lowering the risk of interference with other PS and thus is suitable for faster runs where TLC is not needed. Betulin, having the already mentioned unique structure, will prolong analysis times since it elutes much later than the other sterols. Both cholestane and betulin have substantial chemical and structural differences with common PS analytes, thus their behaviour in certain methods can be different from those of PS e.g., requiring determination of FID (flame ionization detector) response factors, the same not being necessary with 5β-cholestan-3α-ol or 5α-cholestan-3β-ol [237]. The latter ones are hydrogenation products of cholesterol but should be avoided in analyses where cholesterol species are relevant. Even so, epicoprostanol has a good PS-like structure, elutes early, does not occur in plants and can still be used for cholesterol samples, thus being generally a solid choice.

Analysis of PS conjugates and oxidation products has no definite rule of thumb, mostly due to a lack of commercially available standards. POP determinations have used normal [141,150] and deuterated COP [238] standards as well as synthesised POP standards and their deuterated counterparts [149,239]. The latter group showed improved results in terms of sensitivity and detection limits in serum determinations. Since many COP standards are available in trustworthy laboratories (already discussed by other authors e.g., Avanti Polar Lipids) [200] their use as IS in PS determinations is justified, however, depending on the analytical conditions, partial or complete overlapping of COP and POP cannot be overridden e.g., overlapping of 7α-OH-campesterol with 19-OH-cholesterol IS was reported in GC [96]. In many other specific sterol analyses, synthesis of standards becomes necessary, including SEs [107], oxidised SE [240], disteryl ethers [241] and secosterols [242]. SGs have a few commercially available standards, mostly of naturally abundant species (cholesteryl, sitosteryl, campesteryl, and stigmasteryl glucoside), but pure SG can also be acquired from SG standard mixtures (e.g., Matreya LLC) or selected food sources [48]. It should be noted that evaluation of deuterated and non-deuterated standards can be problematic and will require nuclear magnetic resonance (NMR) analyses for definite confirmation of purity and structure, which is mandatory especially if specific stereochemistry is implied in the analysis e.g., separation and characterization of diastereomers [240].

### 6.2. Gas Chromatography (GC) Analysis of PS and PS Esters

GC coupled to mass spectrometry (GC-MS) or flame ionization detector (GC-FID) are the most commonly used methods for the analysis of derivatized PS [223,243,244,245,246]. In the case of PS conjugates, a previous hydrolysis prior to derivatization is required and thus only information about the sterol moiety is obtained. Even so, SG [247] and phenylpropanoid conjugates [248] can also be readily analysed by GC in their silylated form. 

GC-FID is a near-universal-response detector for carbon-containing compounds with only a slight structure dependence, which allows a single standard to be used for the calibration of mixtures (relative percent compositions are achieved straight from the detector responses for all observed peaks without using multiple response factors) [249]. GC-MS has the advantage of allowing an unequivocal identification based on the well-established fragmentation patterns of TMS-PS (trimethylsilylated-PS), which can be compared with database mass spectra (e.g., NIST mass spectral libraries) and existing literature information [250].

**Figure 8:**
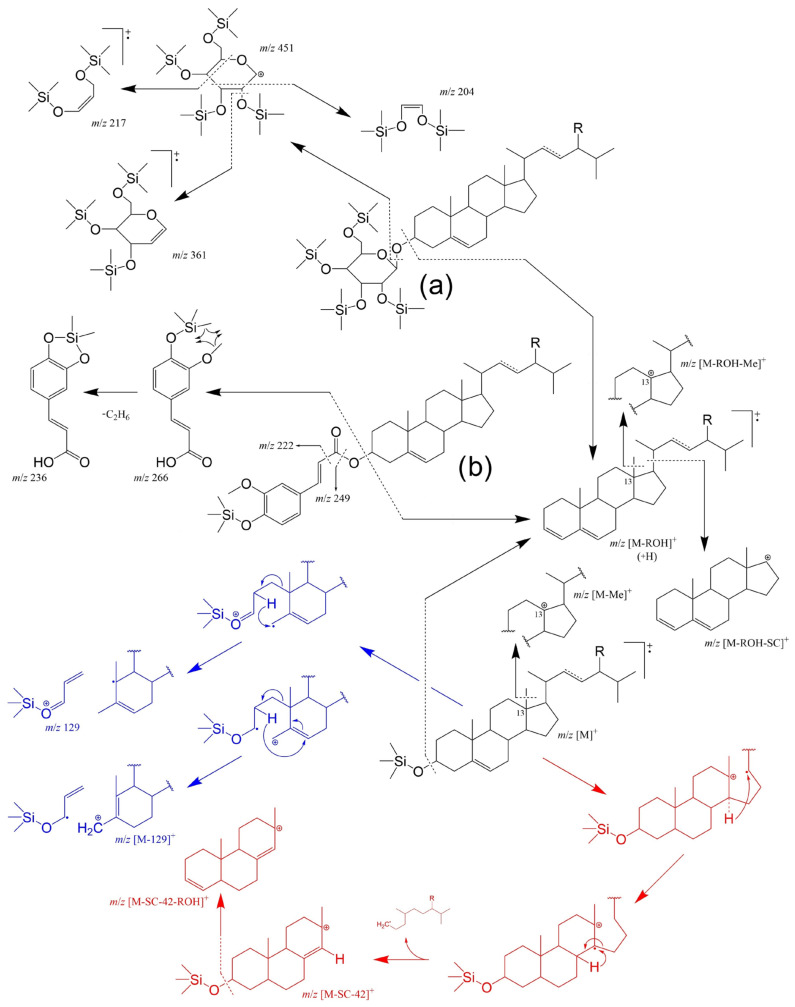
The origins of principal fragments in EI-MS spectra for the most common Δ^5^ free and conjugated sterols, where R-H: cholesterol, R-CH_3_: campesterol, R-CH_3_ with C22=C23: brassicasterol, R-CH_2_CH_3_: sitosterol, and R-CH_2_CH_3_ with C22=C23: stigmasterol. Characteristic fragmentation for the free Δ^5^-PS and corresponding saturated counterparts are marked in blue and red, respectively. In the example of the (**a**) tetra-TMS-steryl-3-β-d-glucopyranoside, being the cleavage of the steryl bond normally accompanied by a charge retention on the sterol moiety leading to *m*/*z* [M – ROH + H]^+^ [247]. The same trend can be observed for (**b**) TMS-steryl ferrulate example [248]. EI-MS of SG TMS ether normally gives a very weak *m*/*z* [M]^+^ and the spectra is dominated by ions from the hexose TMS ether moiety (*m*/*z* 451, 361, 217, 204 in the example) [250].

GC-MS is well suited for structural identification and quantification (with calibration) in general. As such, using GC-FID and GC-MS to complement each other is not uncommon [251,252,253]. The typical fragmentation of the different TMS-PS-derivatives under GC-Electron-impact (EI) mass spectrometry conditions are resumed in Figure 8. Common features of mass spectra fragmentation of free TMS-PS include the observation of the molecular ion M^+^ (with *m*/*z* value equal to the mass of the sterol +72 Da from the silyl group, e.g., *m*/*z* 486 for sitosterol); the fragment ion [M − ROH]^+^ (R = silyl group) formed by the loss of trimethylsilanol; the fragment ions [M − Me]^+^ and [M – ROH − Me]^+^ formed by the loss of the C13 methyl group and combined loss of the methyl group plus trimethylsilanol, respectively; and the product ion [M – ROH − SC]^+^ from the loss of the unsaturated side chain (SC) combined with loss of trimethylsilanol [254]. PS bearing saturated SCs follow different mechanisms depending on the position of the double bond. 

The Δ^5^-steryl TMS ethers also provide characteristic fragment ions at *m*/*z* 129^+^ and at *m*/*z* [M − 129]^+^ involving the loss of the TMS-group together with C1, C2 and C3 of the sterol A-ring (Figure 8 in blue). It must be mentioned that these ions are also seen in Δ^5^-sterols possessing a 4β-methyl or a 4,4-dimethyl grouping, but sterols with mono-unsaturation at other locations in the B/C-rings do not yield these two ions. A Δ^5,7^- or Δ^5,8^-sterol TMS ether will provide a similar fragmentation but with loss of an additional two hydrogens, which results in strong signals at *m*/*z* 131 and the corresponding *m*/*z* [M − 131]^+^. A characteristic fragment of completely saturated homologs can be found at *m*/*z* 215 ([M – SC – 42 − ROH]^+^ in Figure 8). Intermediary [M – SC − 42]^+^ (*m*/*z* 305) can also be observed [254]. 

Following a similar concept, determination of free OPS as TMS ethers is also easily achieved by GC-MS and/or GC-FID, with [M]^+^ being the characteristic fragments for the 7-keto and 5,6-α/β-epoxy, [M – 90]^+^ for the 7-α/β-hydroxy, and [M – 90 – 71]^+^ for triol derivatives [102,243,246,251]. However, special attention should be paid to the various existing α/β isomers (namely their retention times and general sequence) and/or non-hydroxylated PS degradation products (e.g., steradienes/trienes and their patterns) [92]. In the case of analysis of the steryl conjugates (without hydrolyses), these are relatively non-volatile and can also be thermally labile depending on the unsaturation degree of the acyl moiety. As such, there are more suitable methods for effective SE profiling, particularly if the exact pairings of sterol and fatty acid moieties are needed (see next section). Still, GC-MS was shown to determine intact PS fatty acid esters in samples, such as edible oils, without saponification and/or extraction, resulting in minimal analysis time [255]. On a final note, achieving a good resolution of PS components is a key point not only for an effective separation of OPS isomers and/or other PS, but it can also have perks in the case of non-selective detectors such as FID, which can be particularly relevant for substances that have no pure standards available. The best separation of target PS can be achieved via multidimensional approaches, including classical (e.g., GC-GC [256] or LC-GC [225]) or comprehensive 2D chromatography (e.g., GCxGC [257]).

### 6.3. Liquid Chromatography-Mass Spectrometry (LC-MS) and Direct Injection MS^(n)^ Approaches for the Analysis of Sterol Conjugates

Liquid chromatography coupled to mass spectrometry (LC-MS) and direct infusion (DI) mass spectrometry (DI-MS) approaches have been used for the identification and quantification of sterol conjugates, with the advantage of requiring no derivatization or hydrolyses steps as needed in GC. Both lipid extracts or the fraction obtained by SPE or TLC can be analysed directly by LC-MS or DI-MS. These approaches can provide information on PS conjugates, namely on the specific pairings of sterols and fatty acids in fatty acid phytosterol esters (FAPEs), on PS and glucoside moieties in SGs and both FA and sugar moieties in ASG. The FAPEs can be analysed by ESI-MS in positive ion mode by the presence of lithium or ammonium adducts, such as [M + Li]^+^ [258] or [M + NH_4_]^+^ [259,260] adducts, respectively. The FAPEs were also assessed using LC-APPI (atmospheric pressure photoionization)-MS [261] or C8-LC-APCI (atmospheric-pressure chemical ionization)-MS [262] approaches. The [M + H]^+^ ions were observed with the main fragmentation pathways corresponding to the loss of the fatty acyl chain with the formation of abundant [M − FA + H]^+^ ions. In-source fragmentations were reported to occur for stanyl FAPE, displaying abundant [M − FA + H]^+^ ions, while [M + H]^+^, [M − FA + H]^+^ (corresponding to the sterol moiety) and [FA + H]^+^ were observed in the case of stanols with unsaturated FA moiety [262]. This has the advantage of allowing the identification by mass spectrometry fingerprinting, both for the fatty acyl moiety and the sterol moiety of the steryl esters. Regardless, several approaches have been described for the [M − FA + H]^+^-based detection of mixtures of steryl/stanyl FAPEs, which can be very challenging with close *m*/*z* differences between the saturated and unsaturated counterparts [263,264,265]. Thus, multi-reaction monitoring (MRM) approaches can be used to discriminate and quantify isomeric species [262]. Oxidized FAPEs can also be analysed as lithium adducts [M + Li]^+^ in LC-MS, which can be advantageous, since some FAPE species were reported to be less susceptible to form ammonium adducts (7-oxositosteryl oleate and 7β-hydroxysitosteryl oleate) [258]. Given that both the sterol and fatty acid moieties can be oxidized, tandem mass spectrometry (MS/MS) analysis offers the advantage of monitoring the modified counterpart by the assignment of the specific product ions, [sterol moiety + Li]^+^ and [FA + Li]^+^, resulting from the ester bond cleavage.

**Figure 9:**
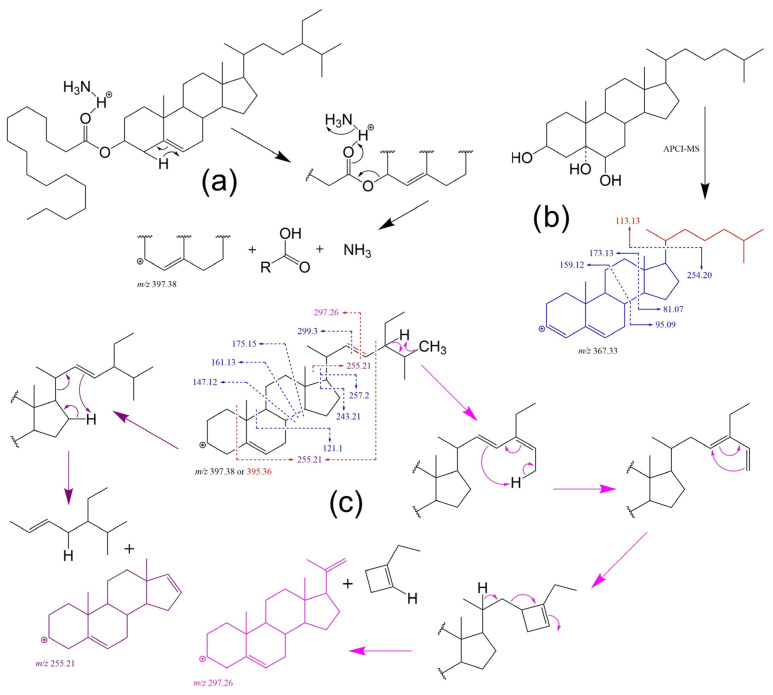
Examples of some fragmentation patterns and mechanisms in ESI-MS-based analyses: (**a**) expected FAPE (sitosteryl palmitate) behaviour in ESI mode as ammonium adduct [278]; (**b**) proposed 5,6-dihydroxycholesterol fragmentation patterns for the steradiene-type precursor ion of *m*/*z* [M + H – 3H_2_O]^+^ in APCI-MS tandem experiment [274]; and (**c**) reported MS/MS patterns for sitosterol (blue) and stigmasterol (red) of the precursor ion *m*/*z* [M + H – H_2_O]^+^ with proposed mechanisms for the formation of characteristic ions of stigmasterol (in purple where *m*/*z* 255.21 can arise from both, side-chain loss and/or partial cleavage of the A ring and side-chain) [268].

Multi-reaction monitoring (MRM) was also used in the direct infusion of ESI-MS approaches. The detections of transitions of ammoniated molecular ions to [M − (FA + NH_3_)]^+^ (Figure 9) were reported in quantitative analyses of normal FAPEs via DI-ESI-MS/MS [266,267,268], i.e., without chromatographic pre-separation, which results in a higher throughput. The approach was also found to be applicable in SG and ASG determination [267]. However, even though this results in a more accurate quantification, direct MS still suffers from ion suppression similarly to LC-MS [269] and cannot distinguish certain isomers (e.g., stigmasterol and Δ^5^-avenasterol) [270].

ESI-based analysis is less suitable for free PS, due to its low-ionization efficiency, However, APCI-MS was found to be more reliable in terms of ion intensity and consistency, with the most abundant ions assigned to [M + H − H_2_O]^+^ [271]. The same [M + H − H_2_O]^+^ trend is observed for SOPs like 7-hydroxy and 5,6-epoxy derivatives, whereas 5,6-dihydroxy (triol) leads to [M + H − 2H_2_O]^+^ and 7-keto results in protonated ion [M + H]^+^ without the loss of the molecule [272,273,274]. Considering the good resolution of the chromatographic separation, LODs (limits of detection) and LLOQs (lower limits of quantitation) achievable in LC, these approaches can be ideal for low-level PS determination, such as in biological samples [161,229], certain beverages/feed [275,276] or in omics profiling analyses including PS [259,277].

### 6.4. Nuclear Magnetic Resonance (NMR)

**Figure 10:**
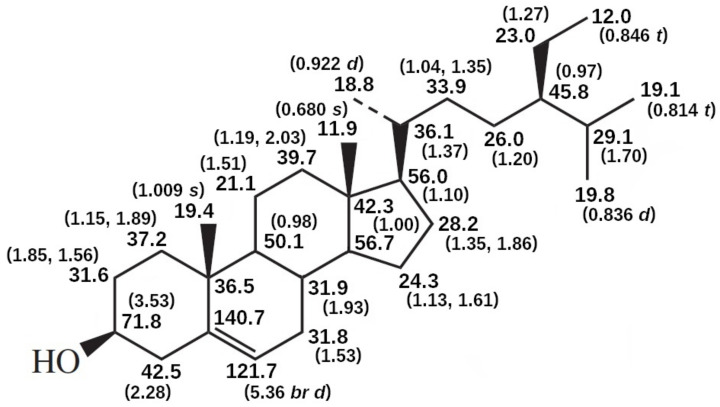
^13^C and ^1^H NMR spectral data of sitosterol in CDCI_3_ [279].

The ^1^H NMR spectrum of a compound provides structural information directly from the presence of specific signals and their relative intensities, while ^13^C NMR spectroscopy offers great sensitivity of chemical shifts to structural changes and the possibility to examine each carbon atom in the molecule individually (Figure 10). Given the variety of PS in natural sources, which differ from one another merely by the presence or absence of unsaturated bonds and alkyl substituents and their locations in the side chain at C-17 position, NMR is perhaps one of the most powerful means for their structural identification (e.g., sitosterol NMR profile in Figure 10). It is also convenient to have several sources of compiled information on NMR data for sterols that can be compared to standards and samples [42] (pp. 197–253).

Chemical shift rules for allylic carbons, homoallylic carbons and olefinic carbons of sterols, presented by Tsuda and Schroepfer 1979 [280], can also be of great use as an initial guideline, as well as the reported NMR substituent-induced chemical shift (SCS) experiments [281,282,283,284]. NMR complementary analysis can be especially useful in synthesised standards and/or sample elucidation of complex structures/isomers such as steryl glucosides [283,285] steryl galactosides [49], acylated steryl glucosides [286], disteryl ethers [115] and oxidation products, including both free sterols [287] and SEs [240]. In terms of using NMR as the base of a PS analysis, even though there is a need of further optimization, ^1^H NMR combined with partial least squares (chemometric model) was shown to have advantages in minimal sample preparation for rapid (5 min) determination of main sterols in vegetable oils [288].

### 6.5. X-ray Diffraction (XRD)

X-ray diffraction (XRD) is based on the scattering of X-ray photons by atoms in a periodic lattice. The scattered monochromatic X-rays that are in phase give constructive interference. In accordance with Bragg’s Law, by measuring the angles under which the constructively interfering X-rays leave the crystal, the interplanar spacings of every single crystallographic phase can be determined. In the case of free PS, XRD can be a useful tool for complementary structural sample characterization, even though the interpretation of the results can be challenging even in relatively pure standards due to innate polymorphism e.g., for sitosterol there are three different pseudopolymorphic crystal forms with different water contents (anhydrous, hemihydrated, and monohydrated crystal forms) and the quantitative determination of hemihydrated and monohydrated crystals according to the XRD results can be difficult because of the preferred orientation of needle-shaped and plate-like crystals [289]. Evaluation of physicochemical characteristics of PE in terms of crystallization behaviour [290], especially during thermal changes, can result in useful information for optimization of PE-based food matrices for properties, such as spreadability, hardness, appearance, and even organoleptic changes [291]. In this area, small angle X-ray scattering (SAXS) and wide-angle X-ray diffraction (WAXD) data, unfortunately extremely scarce, can bring crucial elucidations e.g., chain length and the degree of unsaturation of the fatty acid influence on nanoscale crystal structures formed by the PEs [292].

## 7. Applications of PS

### 7.1. Biologic Activity and Possible Pharmacological Applications 

Besides the well-known hypocholesterolemic activity, as reported in the introduction and metabolism sections, and discussed in the next segment, PS have been the target of many studies on possible biological activities, which have been mostly focused on sitosterol and its derivatives (Table 2). However, amounting information on bioactivity and properties of stigmasterol and stigmasterol-based products was reported by a recent review [293]. The target PS under study vary greatly, ranging from pure commercial standards (e.g., sitosterol Sigma Aldrich standard) to sterol-rich extracts, and even mixed PS products (Table 2). The importance of in vivo data should be outlined since poor bioavailability and pharmacokinetic properties of PS make in vitro studies, based on cultured cells representing systemic tissues and organs, less reliable. However, this limitation has been addressed with the development of different delivery systems for sitosterol, even though, to our current knowledge, none of them have yet reached clinical trials [294,295,296,297,298,299]. Aside from the activities summarized in Table 2, PS were reviewed several times regarding their possible anticancer effects, presenting significant evidence in support of the claim [300,301,302,303]; sitosterol was even shown to have synergistic effects with several known chemotherapeutic drugs [304]. PS consumption has also been linked to lower risks for several types of cancer in epidemiological studies, including colon [305], colorectal [306,307], stomach [308], lung [309], breast [310,311], endometrial [312] and ovarian [313]. The detailed theory on possible mechanisms involved in PS action on cancer cells, such as sphingomyelin turnover, ceramide formation, and liver X receptor activation, are discussed elsewhere [304,314].

### 7.2. Prevention and Control of Hypercholesterolemia

Undoubtedly the paradigmatic indication of PS-based products is related to their hypocholesterolemic activity, and the hypothesized involved mechanisms were already briefly discussed in the metabolism section (Figure 7). This subject has been extensively studied, thus justifying the health promoting claims and dozens of available PS products [13]. However, considering all the available information, there is still a great deal of controversy, particularly in terms of the effects of increased PS plasma levels on the risk and development of cardiovascular diseases, especially when considering controversial epidemiologic studies, where PS plasma concentrations are associated with cardiovascular events [330,331,332,333]. Disparity in the results across reviewed studies can be explained by differences in study design, e.g., plasma PS measurements without evaluation of PS consumption or ignoring ABCG5/8 genetic polymorphisms [168]. In general, as discussed previously, PS have poor bioavailability and, to some degree, many approaches to PS products include structural modifications, usually based on hydrogenation and/or esterification, which has been greatly explored since the 90s. The fact remains that hydrogenated PS (stanols) are coprostanol-like derivatives, and thus their excretion is enhanced when compared to their unsaturated counterparts, further supporting their minimal absorbability. In fact, the conversion of cholesterol into coprostanol by probiotic species is another approach discussed as an alternative to manage hypercholesterolaemia [334]. Additionally, the active forms of plant PS esters are their free counterparts [335] since their hydrolysis in the intestine is required for the cholesterol lowering effects [336]. As such, the hypocholesterolemic activity of PS is at least partially dependent on the availability of the hydroxyl group [337,338]. This is further corroborated by Chung et al. (2008), who reported modified sitosterol esters with enhanced hydrophilicity or lipophilicity to have comparable cholesterol blood lowering effects and only having the solubility advantage vs. free sitosterol [339]. Finally, the hypocholestrolemic effect is mostly warranted as a result of cholesterol displacement during the enterocytic transport and reduction of total intestinal cholesterol uptake [340], which is corroborated by the report that PEs only reduce free cholesterol availability for intestinal absorption when added to a meal in healthy subjects [341]. Nonetheless, functional foods fortified with PS esters are a viable option for non-prescription lowering of plasma LDL and total cholesterol in the management of the associated risk and development of cardiovascular diseases and related cardiovascular events with approved safety and health claims [13,139].

### 7.3. Steroid Production

**Figure 11:**
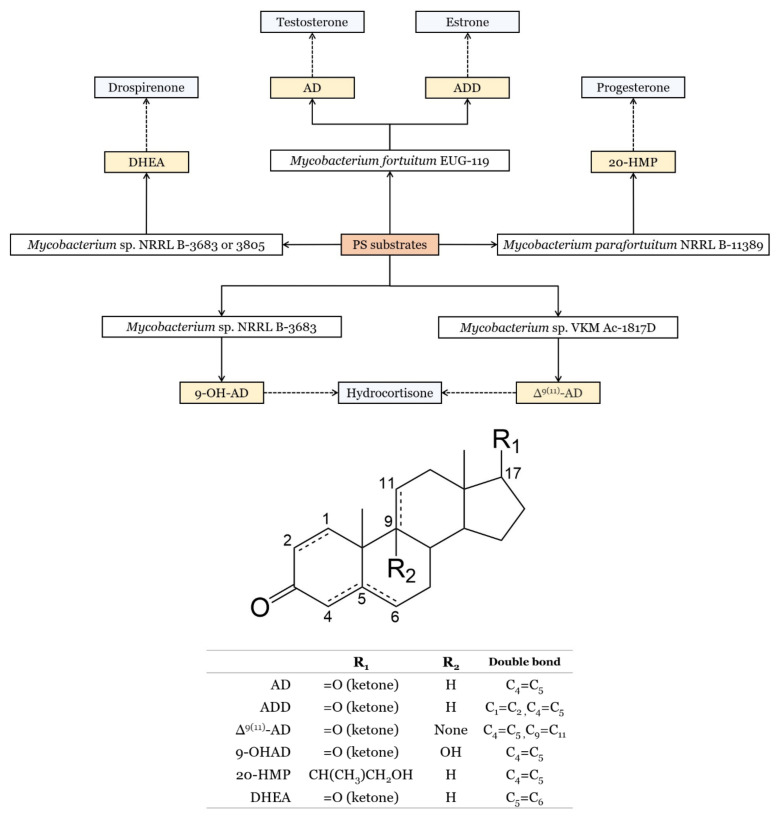
Examples of synthons with respective strains used for their production, based on patented processes [19,20,21,22,28]. Synthons and target representative compounds of respective steroid-drug type are represented in yellow and light blue, respectively. Dash lines refer to chemical steps that lead to the final product. AD: androstenedione, ADD: 1,4-androstadiene-3,17-dione, Δ^9(11)^-AD: androst-4,9(11)-dien-3,17-dione, 9-OHAD: 9α-hydroxy-4-androstene-3,17-dione, 20-HMP: 20-hydroxymethylpregna-1,4-dien-3-one, DHEA: dehydroepiandrosterone.

As already mentioned, steroid chemical synthesis with PS as the starting material goes back to even earlier dates than the trendy hypocholesterolemic effect [14]. However, at present, PS also emerge as cost-effective substrates for optimized bioconversion into steroid intermediates (known as synthons) by genetically engineered strains [17,342]. The concept of steroid biotransformation is far from being new (1950s) [343] but considering that steroid-based drugs are one of the highest marketed categories of pharmaceuticals [342,344], the possibility of bypassing classical steroid synthesis approaches or direct extraction from plant/animal sources makes this a growing area of industrial biotechnology, which is also reflected in several patented works (Figure 11) [19,20,21,22,28]. In fact, large-scale industrial PS bioconversion to key androstane-type synthons has already been introduced in Germany, USA, China, India, among others [17]. Out of all studied microorganisms, *Mycobacterium* species were found the most effective in PS-based biosynthesis of steroidal hormones and intermediates [16].

With preferential cheap raw materials from already discussed sources, cholesterol, sitosterol, stigmasterol, campesterol, and brassicasterol can be considered the main substrates. Products of PS biotransformation can include C-19 synthons such as androstenedione (AD) and 1,4-androstadiene-3,17-dione (ADD), and C-22 derivatives such as 20-hydroxymethylpregna-1,4-dien-3-one (20-HMP), all of which are a staple in chemical synthesis of several key steroids, including sex hormones, anabolic steroids, and adrenocortical hormones [17,343]. In general terms, the main process comprises a selective sidechain cleavage of the sterol molecule and the transformation of the 3-β-ol-5,6-dehydro structure of the steroid nucleus to a 3-keto-4-ene via catabolic enzymatic systems, such as in sitosterol biotransformation to AD by *Mycobacterium* sp. NRRL B-3805, described to involve 11 catabolic enzymes in a 14-consecutive-step pathway [345]. Single-step conversions, such as for testosterone [346] and boldenone [347], have also been described but, in general, most methods will require additional steps, e.g., chemical protection/deprotection of 3-β-OH functionality and/or final chemical steps [342,343]. The recombinant DNA strategies involved in the main steroid bioprocesses were reviewed elsewhere [344].

### 7.4. Other Applications

Other applications of PS-based ingredients include functional foods in direct supplementation for hypercholesterolaemia control, which can be seen as the main application of PS in food industry. However, PS were also considered as food additives per se, for example, as a stabiliser in ready to freeze alcoholic cocktails (stigmasterol-rich E499) [348] or as prospective ingredients in the improvement of the nutritional properties of high saturated and *trans*-fat foods. This includes the use of PS in partial substitution of fat-based creamer/thickeners (e.g., in instant foods and beverages) [26], or as constituents in alternative fat-based food matrices such as oleogels [349]. The latter are a class of a semi-solid functional material comprising a crystal network structure with oil molecules entrapped inside, and their use is not limited to food industry, as they also show promising applicability in pharmaceutical and cosmetic industries [350]. Transparent and firm PS-based gels were shown to be formed in edible oils with a structurant (2–4% *w*/*w*) composed of either sitosterol, cholesterol or dihydrocholesterol, in combination with γ-oryzanol [351]; a patented composition of such oleogels were reported to be suited in consumer goods such as cosmetic or food products [23]. In a similar manner, the PS which self-assemble with monoglycerides in liquid oil were shown to result in oleogels with a texture and thickness closely resembling commercial petrolatum (Vaseline^®^), and thus their use in formulation of personal care products and cosmetics was proposed [352]. PS-based oleogels are also seen as potential release systems [353,354], which can have applications in pharmaceutical formulations of drug delivery, where nanogels were also described as an interesting alternative [355]. In this context, PS have been found to be important constituents in lipid-nanoparticle-based RNA delivery for RNA-based therapeutics [356] or mRNA vaccines [357], as absorption enhancers in topical formulations (β-sitosterol [358] and β-sitosterol β-d-glucoside [359]), and in solutions for parental administration in the form of PS and PS stanol etoxylates. The latter were reported to have equivalent or better solubilizing capacities than cholesterol-based analogues or classical non-ionic surfactants, while being equally or less lytic, particularly to erythrocytes [24,25]. Finally, PS have also been considered as feed additives in poultry and livestock nutrition [360].

## 8. Production of PS

### 8.1. PS Mixture

Large-scale industrial isolation of plant sterols (PS) is based on two main sources of raw materials: vegetable oils from vegetable crops and tall oil from the kraft pulping process. In the first case PS are not normally recovered directly from the crude or refined oil, which has already been outlined as one of the richest sources of PS, but from the deodorizer distillate (DD), a by-product of crude oil refining. Tall oil is also a by-product, but from the softwood kraft pulping process of the pulping industry, which relates to tall oil pitch and tall oil soap. The concentration of free PS in crude tall oil has been reported to be in the range of 2–12.7% [361] which is comparable to DD values of 2–15% free PS and 0–5% PS esters [31]. Nonetheless, in both cases, PS production is related to post-production materials and thus the yields are normally quite low. However, this represents a source of extra profit for manufacturers e.g., 2500 tonnes of vegetable oil or equivalent volume of coniferous (softwood) trees are required to produce one tonne of PS, with seldom exceptions such as DD of biodiesel production where 54 kg of PS from 1 tonne of distillate can be expected [362].

In line with sustainable concepts, agro-industrial waste is also considered an attractive, non-competing source and several alternative by-products for PS production have been characterized over the years, including spent coffee grounds [363], tomato waste [364], rice bran [365], waste biomass of spruce [366], fruit seeds from agro-industrial waste [367], sugarcane waste [368], and grain processing residues from breweries and distilleries [362]. The side streams from hardwood sulphite pulping may represent an underutilized potential source of PS similar to those already explored from softwood kraft pulping, even though the recovery may be challenging [369]. Nonetheless, a promising method was reported for recovering β-sitosterol (BS) (>90% purity) from bleaching effluent of dissolving *Eucalyptus* pulp production using magnesium (Mg^2+^) - based acid sulphite pulping [370].

Regarding the production methods, PS in raw materials are partially found in ester form and often require a pre-treatment involving the hydrolysis of sterol esters, which may be either performed by contacting the material with water and hydrolysing the esters under pressure at high temperature (200–260 °C), or by contacting the PSE-containing material with sodium or potassium hydroxide (saponification) at 90–120 °C, eventually with added pressure, and under stirring [38]. The separation and concentration of the unsaponifiable fraction from residues and by-products are either based on the difference of solubility between unsaponifiable substances and the soap matrix in selected solvents or solvent mixtures, or on the difference of volatility between volatile unsaponifiables and non-volatile salts or soaps. In the latter case, the separation is performed by high-vacuum distillation/evaporation. The process may have several modifications, depending on the source, but some considerations are universal: pH value, PS and FA content, temperatures at which the PS mixture vaporizes, PS crystallization conditions, possible thermal degradation of PS during prolonged heat programs, and close boiling points of PS and other constituents (e.g., tocopherols) [371]. The latter two points can be considered the most iconic challenges in PS recovery, even though the difference in the boiling points between PS and tocopherols can be easily achieved via an esterification step, which makes the separation by distillation viable [31]. The most notorious high-purity examples for PS recovery methods from vegetable oil-based by-products for low sterol content with a high acid value, and for high sterol content with a low acid value, are the WO2004000979 [31] and WO2004111073A2 [30], respectively. When using tall oil pitch/soap and crude tall oil as the raw starting material, most relevant examples include procedures proposed in patent applications WO2000064924A1 [33], WO2003022865A1 [32], and WO2000015652 [37]. A detailed discussion on mentioned patented processes can be found in the review by Fernandes and Cabral (2007) [371].

### 8.2. Phytosterol Esters

The produced free PS are generally not well suited for most manufacturers to work with since they tend to form sharp needle-like crystals at ambient temperatures that result in a brittle product, and have serious solubility problems for incorporation in edible oils or fats. As such, PS esters are synthetically created by esterifying free PS with relatively high levels of mono and polyunsaturated fatty acids. The synthesis can be made by conventional chemical processes, such as those proposed by Johnson and Johnson [34] or Rasio Benecol [40], or alternative biocatalytic methods, e.g., via lipase activity as described in US20150289534A1 [18] and CN101200754B [36]. The methods for esterification with short-chain fatty acids (e.g., carpic and/or caprylic acids) should also be considered since they are easily incorporated into food, have good organoleptic properties, and show improved stability when compared to classic commercial sterol esters with long-chain and unsaturated fatty acids [35].

In the case of specific products for the control of hypercholesterolaemia, stanol-derived products are preferred, and thus, prior hydrogenation of PS is necessary [39]. The possibility to bypass the chemical reduction steps was shown via engineering food plants to replace normal sterol content with the reduced counterparts, e.g., by incorporating a *Streptomyces*-derived 3-hydroxysteroid oxidase gene [372]. In the latter context, plant genetic modifications play an important role in the production enhancement of commercially valuable compounds. However, improving the production of a specific PS via modulation of enzymes in plant mutants can be very challenging since it can lead to highly selective modifications in sterol profiles with unwanted phenotypic results. A classic example would be the overexpression of SMT2;1 in *A. thaliana*, which prompts the biosynthetic flux in the sitosterol branch of the pathway at the expense of campesterol segment, resulting in a higher sitosterol content. Consequently, low levels of campesterol, which was shown to be a precursor of brassinosteroids (regulators of cell elongation and enlargement in many plants species) [64], lead to typical brassinosteroid-deficient phenotypes with size reduction and impaired fertility [373]. Additionally, excess sterols are linked to toxicity in plants [374,375]. Nonetheless, patented approaches for increasing free PS [29] and PS esters [41] in plants were published.

### 8.3. Sitosterol

Sitosterol is one of the most abundant sterols in plants but, surprisingly, very pure commercially available sitosterol products are rare. The reason goes back to sitosterol coexisting with Δ^5^-sterols with extremely similar molecular structures in complex mixtures with other closely related triterpenoids and lipophilic compounds, all of which hinder an effective separation of pure sitosterol. Additionally, sterol fractions with high percentages of sitosterol are also a rare feature for most PS sources, with exceptions such as rhizomes of *Cimicifuga acerina* (sitosterol being almost the exclusive PS component) [376] and diatome *Asterionella glacialis* (95% of total PS) [377]. However, certain processed raw materials, such as some vegetable oils, can also have very high sitosterol levels, e.g., in avocado oil (≈91.9–94.8% of unsaponifiables) [378]. As such, if very large amounts (grams) of sitosterol are required, it is often necessary to resort to the older classical methods of natural products purification by large-scale alumina or silica-gel column chromatography and fractional crystallization [42] (pp. 283–314). It is possible to consider three general strategies for obtaining gram quantities of individual sitosterol: conversion of stigmasterol through selective hydrogenation or reduction of the Δ^22–23^ alkene with simultaneous protection of the Δ^5–6^ double bond (since stigmasterol is the most accessible and least expensive PS) [287]; isolation of sitosterol from vegetable oils via employing a series of crystallizations (quite cost-effective and sitosterol purity rarely exceeding 70%; a process similar to discussed for general PS production); and purification of existing PS mixtures (over 90%) via silica gel chromatography or Na-Y zeolite (with repeated, time-consuming cycles of column purification). The last approach was somewhat improved using a combination of fractional crystallization, silica gel and Na-Y zeolite chromatography achieving the desired purity under 72 h [379]. As such, it can be concluded that the development of an efficient and cost-effective process for producing high purity sitosterol from suitable sources is still a challenge. Alleged sources should preferably be non-competitive with food chains and available year-round (non-seasonal).

## 9. Final Considerations on Future Trends and Research

Considering all of the above, several topics deserve to be addressed in terms of future approaches for further advancement in PS production and investigation of its rational use/safety and development of functional foods based on PS: (1) search for new non-competing sources such as alternative prospective industrial or agro-industrial by-products (Production, Section 6) and/or new species of starting raw materials, e.g., microalgae [380]; (2) further assessment of long-term effects of oxyphytosterols in different biological models and especially in vivo in healthy and unhealthy subjects [139], which can be in the scope of major emerged areas of cholesterol-derived oxysterol biology, i.e., oxysterol effects on regulation of intracellular cholesterol metabolism, cellular cytotoxicity, sterol efflux from macrophages and activation of nuclear transcription factors [381]; (3) the need for epidemiological data on inter-individual variability and genomic influence in PS consumption [382,383]; (4) find effective strategies in plant gene modification (cell engineering) for simpler production steps, higher yields or specific products, linked to further elucidation of main and secondary biosynthetic pathways [384] and especially genes encoding key enzymes and regulators in key species, e.g., identification of genes encoding SE esterases, which have yet to be reported in plants [385]; (5) safety assessment for PS-based products from genetically modified sources; (6) fill the gap in research on gut microbiota and probiotics biotransformation routes of dietary and functional food, e.g., additional information is needed on the interaction in situ of considered pro-carcinogenic cholesterol metabolites and PS in gastrointestinal cancer etiology, since poor PS absorption corroborates a possible local activity [386], as well as the impact of PS in terms of modulation of the gut microbiota metabolic activity, already tackled by some researchers [387]; (7) further evaluation of the efficacy and safety of PS vs. probiotic-induced cholesterol to coprostanol conversion, in terms of hypercholesterolaemia and cardiovascular diseases control [334]; (8) the deepening of systematic studies on the impact of different food/product matrixes on bioavailability and oxidation of PS/stanols and their esters upon processing and storage (or vice versa: e.g., the biological effects of protein modification by POP) [388]; (9) the development of highly efficient industrial processes for production of food grade hydrophilic PS derivatives without compromising their cholesterol-lowering activity, e.g., natural PS glycosides [389]; and (10) provide new analytical approaches for tertiary POP (e.g., oligomers) as well as oxidized steryl-/stanyl fatty acid esters, and the synthesis and validation of related PS standards. In this regard, mass spectrometry was steadily pushed as a solo quantitative tool with virtually limitless capabilities according to newer technologies, but most works focus on a one-fit approach, and it is necessary to prompt the development of fully validated methods that fall within the acceptance criteria of regulatory guidelines not only in classical analyses, but also in metabolomics [275,390,391,392,393].

## Figures and Tables

**Table 1:** Total PS content range in major food sources, expressed in fresh weight (FW).

Source ^a^	PS Range in FW (mg/100 g) ^a^
Vegetable oils	60–2600
Milling products	130–586
Margarines	40–411
Nuts	22–220
Cereals	35–121
Bakery products	39–90
Fruits and berries	1–75
Vegetables	4–43
Estimated non-vegetarian daily intake ^b^	0.2–0.3 g/day
Recommended daily intake ^c^	2.0 g/day

^a^ The sources and PS range based on Piironen and Lampi (2004) [132]. ^b^ Estimated daily intake is stipulated from Ras et al. (2013) [131]. ^c^ Recommended daily intake refers to the main purpose of lowering total and low-density lipoprotein cholesterol.

**Table 2:** In vivo studies on sitosterol and derivatives supporting different biological activities.

Property	Active Compound(s)	Main Feature	Ref.
Analgesic	β-Sitosterol and β-sitosteryl-β-d-glucoside from leaves of *Mentha cordifolia* Opiz.	300% increase in pain tolerance for sitosterol	[315]
Androgenetic alopecia	β-Sitosterol phyto-vesicles ^1^	Maximum hair follicle density after 21 days	[299]
Angiogenic	β-Sitosterol from *A. Vera*	Enhanced new vessel formation in gerbil brains	[316]
Anticoagulant	Soybean-derived sitosterol	Inhibited k-carrageen-induced thrombus formation	[317]
Antifertility	β-sitosterol from roots of *Barleria prionitis*	Suppression of spermatogenesis: potential male contraceptive	[318]
Anti-inflammatory	Sitosterol from *Justicia gendarussa* Burm F.	Potent activity via histamine, serotonin, bradykinin and prostaglandin release	[319]
Atopic Dermatitis (AD)	β-sitosterol ^2^	AD clinical symptoms such as eczematous erythema and dryness	[320]
Benign prostatic hyperplasia	β-sitosterol ^2^ enriched saw palmetto oil	Inhibition of COX-2 and NF-κB expression.	[321]
Glucose homeostasis	β-Sitosterol from the unripe fruits of *Coccinia grandis*	Enhanced protein expression of PPARγ and glucose transporter 4	[322]
Hepatic inflammation	Sterol/stanol esters	Reduced inflammatory response in liver	[323]
Immunomodulatory	Inmunicín MAYMO^®^ ^3^	Dentritic cell activation and up regulation of IFN-α	[324]
Metabolic syndrome	β-sitosterol ^2^	Decreased plasma insulin concentration and increased insulin sensitivity	[325]
Neuroprotective	β-sitosterol isolated from *G. carpinifolia*	Myelo-protective activities; enhanced cognition and improved motor co-ordination	[326]
Obesity-related chronic inflammation	High-fat diet with PS mixture ^4^	Negative correlation between sitosterol and IL-6 and TNF-α serum levels	[327]
Type-2 diabetes management	β-sitosterol ^2^	Attenuated serine phosphorylation of IRS-1 in adipose tissue	[328]
Wound healing	Synthetic sitosterol derivative ^5^	Inhibitory activity on Na^+^/K^+^-ATPase; substantially improved healing.	[329]

^1^ Complexation with phosphatidyl choline. ^2^ Sitosterol Sigma-Aldrich. ^3^ Spanish PS product with sitosterol as the main component. ^4^ 326 μg of cholesterol, 18.0 μg of sitosterol, 2.85 μg of campesterol, and 5.07 μg of 5α-cholestanol per gram. ^5^ (*E*)-sitosterol-3β,6β-hydroxyimino-*P*-methylbenzyl-4-ene.

## References

[1] Burián R. (1897). Über Sitosterin. Monatsh. Chem..

[2] Anderson R.J., Shriner R.L. (1926). The Phytosterols of Corn Oil. J. Am. Chem. Soc..

[3] Anderson R.J., Shriner R.L., Burr G.O. (1926). The Phytosterols of Wheat Germ Oil. J. Am. Chem. Soc..

[4] Bloch K. (1992). Sterol molecule: Structure, biosynthesis, and function. Steroids.

[5] Schoenheimer R. (1931). New contributions in sterol metabolism. Science.

[6] Peterson D.W. (1951). Effect of soybean sterols in the diet on plasma and liver cholesterol in chicks. Proc. Soc. Exp. Biol. Med..

[7] Pollak O.J. (1953). Reduction of blood cholesterol in man. Circulation.

[8] Moreau R.A., Whitaker B.D., Hicks K.B. (2002). Phytosterols, phytostanols, and their conjugates in foods: Structural diversity, quantitative analysis, and health-promoting uses. Prog. Lipid Res..

[9] Endo A. (2010). A historical perspective on the discovery of statins. Proc. Jpn. Acad. Ser. B Phys. Biol. Sci..

[10] Buț M.-G., Jîtcă G., Imre S., Vari C.E., Ősz B.E., Jîtcă C.-M., Tero-Vescan A. (2023). The Lack of Standardization and Pharmacological Effect Limits the Potential Clinical Usefulness of Phytosterols in Benign Prostatic Hyperplasia. Plants.

[11] Ulbricht C.E. (2016). An Evidence-Based Systematic Review of Beta-Sitosterol, Sitosterol (22,23-dihydrostigmasterol, 24-ethylcholesterol) by the Natural Standard Research Collaboration. J. Diet Suppl..

[12] Moreau R.A., Dutta P.C. (2003). Chapter 7. Plant Sterols in Functional Foods. Phytosterols as Functional Food Components and Nutraceuticals.

[13] Zawistowski J., Jones P. (2015). Regulatory Aspects Related to Plant Sterol and Stanol Supplemented Foods. J. AOAC Int..

[14] Lednicer D. (2011). Chapter 2—Sources of steroids. Steroid Chemistry at a Glance.

[15] Seeman J.I. (2022). Percy Lavon Julian: A man who rose to every occasion. Proc. Natl. Acad. Sci. USA.

[16] Das S., Gopishetty S., Wilson D.B., Sahm H., Stahmann K.-P., Koffas M. (2020). Chapter 12—Steroids. Industrial Microbiology.

[17] Donova M. (2021). Microbial Steroid Production Technologies: Current Trends and Prospects. Microorganisms.

[18] Huang F., Zheng M., Shi W., Xiang X., Shi J., Deng Q., Li W., Wan C. (2015). Method for Preparing Functional Edible Oil Rich in Phytosterol Esters and Diglycerides. U.S. Patent.

[19] Imada Y., Takahashi K. (1980). Process for Producing Steroidal Alcohols. U.S. Patent.

[20] Kazantsev A.V., Savinova T.S., Lukashev N.V., Desyatkin V.G., Dovbnya D.V., Khomutov S.M., Sukhodolskaya G.V., Shutov A.A., Donova M.V., Egorova O.V. (2014). Method of Obtaining Androst-4,9(11)-Dien-3,17-Dione from Phytosterol. RU Patent.

[21] Liu X.Q., Meng H., Yang K. (2013). Method for Preparing Dehydroepiandrosterone by Microbial Fermentation. CN Patent.

[22] Noh S.K., Kim M.K., Yoon W.T., Park K.M., Park S.O. (2002). A Method for Preparation of Androst-4-Ene-3,17-Dione and Androsta-1,4-Diene-3,17-Dione. WO Patent.

[23] Ritter H., van de Sande R.L., Muller V. (2005). Liquid Fatty Component Containing Composition. U.S. Patent.

[24] Söderlind E. (2003). New Use of Ethoxylated Phytosterols and Phytostanols. WO Patent.

[25] Soderlind E. (2004). Use of Ethoxylated Phytosterols and Phytostanols. U.S. Patent.

[26] Veldhuizen Y.S.J., Weisbecker R.T. (2005). Particulate Comprising Phytosterols and Food Compositions Comprising Said Creamer. WO Patent.

[27] Glade T.F.H., Diaz M.A.F., Rojas A.M. (2017). Phytosterol Dispersions. CA Patent.

[28] Zhao X., Liu X., Meng H., Zeng C., Yang F. (2019). Method for the Transformation and Separation of 9α-OH-AD and Methyl Esters of Phytosterols. CN Patent.

[29] Chappel J., Saunders C.A., Wolf F.R., Cuellar R.E. (2003). Method and Composition for Increasing Sterol Accumulation in Higher Plants. EP Patent.

[30] Charlemagne D., Bostyn S., Daguet D. (2004). Sterol Recovering Method. WO Patent.

[31] Czuppon T., Kemeny Z., Kovari E., Recseg K. (2003). Process for Recovery of Plant Sterols from by-Product of Vegetable Oil Refining. WO Patent.

[32] Hamunen A. (2003). Process for the Isolation of Sterols and/or Wax Alcohols from Tall Oil Products. WO Patent.

[33] Hamunen A., Ukkonen K. (2000). Process for the Purification of Sterols from Hydrocarbon Extracts Using Evaporative Fractionation. WO Patent.

[34] Higgins J.D. (2000). Preparation of Sterol and Stanol-Esters. U.S. Patent.

[35] Horlacher P., Hietsch D., Albiez W., Timmermann F., Beck K. (2007). Sterol Esters Having Short-Chained Fatty Acids. WO Patent.

[36] Hu X., Chen H., Jiang M., Dong X., Liu C., Wei F., Zhang Y., Huang F. (2010). Method for Producing Plant Sterol Ester by Immobilized Whole-Cell Enzyme Catalysis in Solvent-Free System. CN Patent.

[37] Huibers D.T.A., Robbins A.M., Sullivan D.H. (2000). Method for Separating Sterols from Tall Oil. WO Patent.

[38] Rohr R., Rohr R., Trujillo-Quijano J.A. (2005). Process for Separating Unsaponifiable Valuable Products from Raw Materials. U.S. Patent.

[39] Wong A., Boguski W.N. (2000). Preparation of Saturated Phytosterols. CA Patent.

[40] Wester I., Ekblom J. (2003). Novel Compositions of Phytosterol and Phytostanol Fatty Acid Esters, Products Containing Them and Processes for Their Preparation. FI Patent.

[41] Zou J., Chen Q. (2007). Increased Phytosterol Content through Overexpression of an Acyl-Coa Sterol Acyltransferase. WO Patent.

[42] Goad L.J., Akihisa T. (1997). Analysis of Sterols.

[43] Ikekawa N., Fujimoto Y., Kadota S., Kikuchi T. (1989). Effective separation of sterol C-24 epimers. J. Chromatogr. A.

[44] Dewick P.M. (2009). The mevalonate and methylerythritol phosphate pathways: Terpenoids and steroids. Medicinal Natural Products. A Biosynthetic Approach.

[45] Axelos M., Péaud-Lenoël C., Loewus F.A., Tanner W. (1982). Steryl Glycosides. Plant Carbohydrates I Intracellular Carbohydrates.

[46] Akiyama H., Ide M., Nagatsuka Y., Sayano T., Nakanishi E., Uemura N., Yuyama K., Yamaguchi Y., Kamiguchi H., Takahashi R. (2020). Glucocerebrosidases catalyze a transgalactosylation reaction that yields a newly-identified brain sterol metabolite, galactosylated cholesterol. J. Biol. Chem..

[47] Hirai Y., Haque M., Yoshida T., Yokota K., Yasuda T., Oguma K. (1995). Unique cholesteryl glucosides in Helicobacter pylori: Composition and structural analysis. J. Bacteriol..

[48] Münger L.H., Boulos S., Nyström L. (2018). UPLC-MS/MS Based Identification of Dietary Steryl Glucosides by Investigation of Corresponding Free Sterols. Front. Chem..

[49] Heinz P., Glomb M.A. (2018). Characterization and Quantitation of Steryl Glycosides in *Solanum melongena*. J. Agric. Food Chem..

[50] Akihisa T., Yasukawa K., Yamaura M., Ukiya M., Kimura Y., Shimizu N., Arai K. (2000). Triterpene alcohol and sterol ferulates from rice bran and their anti-inflammatory effects. J. Agric. Food Chem..

[51] Zhu D., Nyström L. (2015). Differentiation of rice varieties using small bioactive lipids as markers. Eur. J. Lipid Sci. Technol..

[52] Takagi T., Iida T. (1980). Antioxidant for fats and oils from canary seed—Sterol and triterpene alcohol esters of caffeic acid. J. Am. Oil Chem. Soc..

[53] Aladedunye F., Przybylski R., Rudzinska M., Klensporf-Pawlik D. (2013). γ-Oryzanols of North American Wild Rice (*Zizania palustris*). J. Am. Oil Chem. Soc..

[54] Kuzuyama T., Seto H. (2012). Two distinct pathways for essential metabolic precursors for isoprenoid biosynthesis. Proc. Jpn. Acad. Ser. B Phys. Biol. Sci..

[55] Ohyama K., Suzuki M., Kikuchi J., Saito K., Muranaka T. (2009). Dual biosynthetic pathways to phytosterol via cycloartenol and lanosterol in *Arabidopsis*. Proc. Natl. Acad. Sci. USA.

[56] Korber M., Klein I., Daum G. (2017). Steryl ester synthesis, storage and hydrolysis: A contribution to sterol homeostasis. Biochim. Biophys. Acta Mol. Cell Biol. Lipids..

[57] Lara J.A., Burciaga-Monge A., Chávez A., Revés M., Lavilla R., Arró M., Boronat A., Altabella T., Ferrer A. (2018). Identification and Characterization of Sterol Acyltransferases Responsible for Steryl Ester Biosynthesis in Tomato. Front. Plant Sci..

[58] Dyas L., Goad L.J. (1993). Steryl fatty acyl esters in plants. Phytochemistry.

[59] Nes W.D. (2011). Biosynthesis of cholesterol and other sterols. Chem. Rev..

[60] Darnet S., Blary A., Chevalier Q., Schaller H. (2021). Phytosterol Profiles, Genomes and Enzymes—An Overview. Front. Plant Sci..

[61] Grosjean K., Mongrand S., Beney L., Simon-Plas F., Gerbeau-Pissot P. (2015). Differential effect of plant lipids on membrane organization: Specificities of phytosphingolipids and phytosterols. J. Biol. Chem..

[62] Beck J.G., Mathieu D., Loudet C., Buchoux S., Dufourc E.J. (2007). Plant sterols in “rafts”: A better way to regulate membrane thermal shocks. FASEB J..

[63] Grandmougin-Ferjani A., Schuler-Muller I., Hartmann M.A. (1997). Sterol Modulation of the Plasma Membrane H^+^-ATPase Activity from Corn Roots Reconstituted into Soybean. Lipids Plant Physiol..

[64] Yokota T. (1997). The structure, biosynthesis, and function of brassinosteroids. Trends Plant Sci..

[65] Sonawane P.D., Pollier J., Panda S., Szymanski J., Massalha H., Yona M., Unger T., Malitsky S., Arendt P., Pauwels L. (2016). Plant cholesterol biosynthetic pathway overlaps with phytosterol metabolism. Nat. Plants.

[66] Schrick K., Mayer U., Martin G., Bellini C., Kuhnt C., Schmidt J., Jürgens G. (2002). Interactions between sterol biosynthesis genes in embryonic development of *Arabidopsis*. Plant J..

[67] Schrick K., Mayer U., Horrichs A., Kuhnt C., Bellini C., Dangl J., Schmidt J., Jürgens G. (2000). FACKEL is a sterol C-14 reductase required for organized cell division and expansion in *Arabidopsis* embryogenesis. Genes Dev..

[68] Luo M., Xiao Y., Li X., Lu X., Deng W., Li D., Hou L., Hu M., Li Y., Pei Y. (2007). GhDET2, a steroid 5alpha-reductase, plays an important role in cotton fiber cell initiation and elongation. Plant J..

[69] Willemsen V., Friml J., Grebe M., van den Toorn A., Palme K., Scheres B. (2003). Cell polarity and PIN protein positioning in *Arabidopsis* require STEROL METHYLTRANSFERASE1 function. Plant Cell.

[70] Schrick K., Fujioka S., Takatsuto S., Stierhof Y.D., Stransky H., Yoshida S., Jurgens G. (2004). A link between sterol biosynthesis, the cell wall, and cellulose in *Arabidopsis*. Plant J..

[71] Schrick K., Debolt S., Bulone V. (2012). Deciphering the molecular functions of sterols in cellulose biosynthesis. Front. Plant Sci..

[72] Yu L., Fan J., Zhou C., Xu C. (2021). Sterols are required for the coordinated assembly of lipid droplets in developing seeds. Nat. Commun..

[73] Souter M., Topping J., Pullen M., Friml J., Palme K., Hackett R., Grierson D., Lindsey K. (2002). Hydra mutants of *Arabidopsis* are defective in sterol profiles and auxin and ethylene signaling. Plant Cell.

[74] Souter M.A., Pullen M.L., Topping J.F., Zhang X., Lindsey K. (2004). Rescue of defective auxin-mediated gene expression and root meristem function by inhibition of ethylene signalling in sterol biosynthesis mutants of *Arabidopsis*. Planta.

[75] Griebel T., Zeier J. (2010). A role for β-sitosterol to stigmasterol conversion in plant–pathogen interactions. Plant J..

[76] Kopischke M., Westphal L., Schneeberger K., Clark R., Ossowski S., Wewer V., Fuchs R., Landtag J., Hause G., Dörmann P. (2013). Impaired sterol ester synthesis alters the response of *Arabidopsis thaliana* to *Phytophthora infestans*. Plant J..

[77] Wang K., Senthil-Kumar M., Ryu C.-M., Kang L., Mysore K.S. (2012). Phytosterols play a key role in plant innate immunity against bacterial pathogens by regulating nutrient efflux into the apoplast. Plant Physiol..

[78] Kumar M.S., Ali K., Dahuja A., Tyagi A. (2015). Role of phytosterols in drought stress tolerance in rice. Plant Physiol. Biochem..

[79] Kumar M.S., Mawlong I., Ali K., Tyagi A. (2018). Regulation of phytosterol biosynthetic pathway during drought stress in rice. Plant Physiol. Biochem..

[80] Posé D., Castanedo I., Borsani O., Nieto B., Rosado A., Taconnat L., Ferrer A., Dolan L., Valpuesta V., Botella M.A. (2009). Identification of the Arabidopsis dry2/sqe1-5 mutant reveals a central role for sterols in drought tolerance and regulation of reactive oxygen species. Plant J..

[81] Wagatsuma T., Khan M.S.H., Watanabe T., Maejima E., Sekimoto H., Yokota T., Nakano T., Toyomasu T., Tawaraya K., Koyama H. (2014). Higher sterol content regulated by CYP51 with concomitant lower phospholipid content in membranes is a common strategy for aluminium tolerance in several plant species. J. Exp. Bot..

[82] Takshak S., Agrawal S.B. (2015). Alterations in metabolite profile and free radical scavenging activities of *Withania somnifera* leaf and root extracts under supplemental ultraviolet-B radiation. Acta Physiol. Plant..

[83] Kuczyńska A., Cardenia V., Ogrodowicz P., Kempa M., Rodriguez-Estrada M.T., Mikołajczak K. (2019). Effects of multiple abiotic stresses on lipids and sterols profile in barley leaves (*Hordeum vulgare* L.). Plant Physiol. Biochem..

[84] Mishra M.K., Singh G., Tiwari S., Singh R., Kumari N., Misra P. (2015). Characterization of *Arabidopsis* sterol glycosyltransferase TTG15/UGT80B1 role during freeze and heat stress. Plant Signal. Behav..

[85] Rogowska A., Szakiel A. (2020). The role of sterols in plant response to abiotic stress. Phytochem. Rev..

[86] Senthil-Kumar M., Wang K., Mysore K.S. (2013). AtCYP710A1 gene-mediated stigmasterol production plays a role in imparting temperature stress tolerance in *Arabidopsis thaliana*. Plant Signal. Behav..

[87] Valitova J., Renkova A., Mukhitova F., Dmitrieva S., Beckett R.P., Minibayeva F.V. (2019). Membrane sterols and genes of sterol biosynthesis are involved in the response of *Triticum aestivum* seedlings to cold stress. Plant Physiol. Biochem..

[88] Feng S., Belwal T., Li L., Limwachiranon J., Liu X., Luo Z. (2020). Phytosterols and their derivatives: Potential health-promoting uses against lipid metabolism and associated diseases, mechanism, and safety issues. Compr. Rev. Food Sci. Food Saf..

[89] Jie F., Yang X., Wu L., Wang M., Lu B. (2022). Linking phytosterols and oxyphytosterols from food to brain health: Origins, effects, and underlying mechanisms. Crit. Rev. Food Sci. Nutr..

[90] Dutta P.C., Przybylski R., Eskin M.N., Appelqvist L.-A., Erickson M.D. (2007). Formation, analysis, and health effects of oxidized sterols in frying fat. Deep Frying.

[91] Hovenkamp E., Demonty I., Plat J., Lütjohann D., Mensink R.P., Trautwein E.A. (2008). Biological effects of oxidized phytosterols: A review of the current knowledge. Prog. Lipid Res..

[92] Hu Y., Huang W., Li M., Wang M., Zhao Y., Xu T., Zhang L., Lu B., He Y. (2017). Metal ions accelerated phytosterol thermal degradation on Ring A & Ring B of steroid nucleus in oils. Food Res. Int..

[93] Smith L.L. (1981). Chapter IV—Initial events of autoxidation. Cholesterol Autoxidation.

[94] Conchillo A., Cercaci L., Ansorena D., Rodriguez-Estrada M.T., Lercker G., Astiasarán I. (2005). Levels of phytosterol oxides in enriched and nonenriched spreads: Application of a thin-layer chromatography-gas chromatography methodology. J. Agric. Food Chem..

[95] Johnsson L., Dutta P.C. (2006). Determination of phytosterol oxides in some food products by using an optimized transesterification method. Food Chem..

[96] Soupas L., Juntunen L., Säynäjoki S., Lampi A.M., Piironen V. (2004). GC-MS method for characterisation and quantification of sitostanol oxidation products. J. Am. Oil Chem. Soc..

[97] Lengyel J., Rimarcík J., Vagánek A., Fedor J., Lukeš V., Klein E. (2012). Oxidation of sterols: Energetics of C–H and O–H bond cleavage. Food Chem..

[98] Martins I.R., Onuki J., Miyamoto S., Uemi M. (2020). Characterization of oxyphytosterols generated by β-sitosterol ozonization. Arch. Biochem. Biophys..

[99] Johnsson L., Dutta P.C. (2003). Characterization of side-chain oxidation products of sitosterol and campesterol by chromatographic and spectroscopic methods. J. Am. Oil Chem. Soc..

[100] Johnsson L., Andersson R.E., Dutta P.C. (2003). Side-chain autoxidation of stigmasterol and analysis of a mixture of phytosterol oxidation products by chromatographic and spectroscopic methods. J. Am. Oil Chem. Soc..

[101] Giuffrida F., Destaillats F., Robert F., Skibsted L.H., Dionisi F. (2004). Formation and hydrolysis of triacylglycerol and sterols epoxides: Role of unsaturated triacylglycerol peroxyl radicals. Free Radic. Biol. Med..

[102] Derewiaka D., Obiedzinski M. (2012). Phytosterol oxides content in selected thermally processed products. Eur. Food Res. Technol..

[103] Finocchiaro E.T., Richardson T. (1983). Sterol Oxides in Foodstuffs: A Review. J. Food Prot..

[104] Soupas L., Huikko L., Lampi A.M., Piironen V. (2007). Pan-frying may induce phytosterol oxidation. Food Chem..

[105] Soupas L., Juntunen L., Lampi A.M., Piironen V. (2004). Effects of sterol structure, temperature, and lipid medium on phytosterol oxidation. J. Agric. Food Chem..

[106] Zhang X., Julien-David D., Miesch M., Raul F., Geoffroy P., Aoude-Werner D., Ennahar S., Marchioni E. (2006). Quantitative analysis of beta-sitosterol oxides induced in vegetable oils by natural sunlight, artificially generated light, and irradiation. J. Agric. Food Chem..

[107] Chaijan M., Panpipat W. (2020). Instability of β-sitosteryl oleate and β-sitosterol loaded in oil-in-water emulsion. NFS J..

[108] Kasprzak M., Rudzińska M., Kmiecik D., Przybylski R., Olejnik A. (2020). Acyl moiety and temperature affects thermo-oxidative degradation of steryl esters. Cytotoxicity of the degradation products. Food Chem. Toxicol..

[109] Miyamoto S., Limam R.S., Inague A., Viviani L.G. (2021). Electrophilic oxysterols: Generation, measurement and protein modification. Free Radic. Res..

[110] Zschau W. (2001). Bleaching of edible fats and oils IX. Legal and analytical aspects of bleaching from the working group “Technologies of industrial extraction and processing of edible fats”. Eur. J. Lipid Sci. Technol..

[111] Schulte E., Weber N. (1987). Analysis of disteryl ethers. Lipids.

[112] Rudzinska M., Przybylski R., Zhao Y.Y., Curtis J.M. (2010). Sitosterol thermo-oxidative degradation leads to the formation of dimers, trimers and oligomers: A study using combined size exclusion chromatography/mass spectrometry. Lipids.

[113] Struijs K., Lampi A.M., Ollilainen V., Piironen V. (2010). Dimer formation during the thermo-oxidation of stigmasterol. Eur. Food Res. Technol..

[114] Zmysłowski A., Sitkowski J., Bus K., Ofiara K., Szterk A. (2020). Synthesis and search for 3β,3′β-disteryl ethers after high-temperature treatment of sterol-rich samples. Food Chem..

[115] Zmysłowski A., Sitkowski J., Bus K., Michalska K., Szterk A. (2021). Synthesis of Oxidized 3β,3′β-Disteryl Ethers and Search after High-Temperature Treatment of Sterol-Rich Samples. Int. J. Mol. Sci..

[116] Lehtonen M., Lampi A.-M., Agalga F., Struijs K., Piironen V. (2011). The effects of acyl moiety and temperature on the polymerization of sterols. Eur. J. Lipid Sci. Technol..

[117] He W.S., Zhu H., Chen Z.Y. (2018). Plant Sterols: Chemical and Enzymatic Structural Modifications and Effects on Their Cholesterol-Lowering Activity. J. Agric. Food Chem..

[118] Ng A.W., Lukic T., Pritchard P.H., Wasan K.M. (2003). Development of novel water-soluble phytostanol analogs: Disodium ascorbyl phytostanyl phosphates (FM-VP4): Preclinical pharmacology, pharmacokinetics and toxicology. Cardiovasc. Drug Rev..

[119] Xia X., Ren M., He W.S., Jia C., Zhang X. (2022). The preparation of phytosteryl succinyl sucrose esters and improvement of their water solubility and emulsifying properties. Food Chem..

[120] Folmer B.M. (2003). Sterol surfactants: From synthesis to applications. Adv. Colloid Interface Sci..

[121] Holmberg K. (2001). Natural surfactants. COCIS.

[122] Madawala S.R.P., Andersson R.E., Jastrebova J.A., Almeida M., Dutta P.C. (2012). Phytosterol and α-Lipoic Acid Conjugates: Synthesis, Free Radical Scavenging Capacity and RP-LC-MS-APCI Analysis. Polish J. Food Nutr. Sci..

[123] Wang Z., Hwang S.H., Lim S.S. (2015). Lipophilization of phenolic acids with phytosterols by a chemoenzymatic method to improve their antioxidant activities. Eur. J. Lipid Sci. Technol..

[124] Tan Z., Shahidi F. (2011). Chemoenzymatic synthesis of phytosteryl ferulates and evaluation of their antioxidant activity. J. Agric. Food Chem..

[125] Rabiej-Kozioł D., Krzemiński M.P., Szydłowska-Czerniak A. (2021). Steryl Sinapate as a New Antioxidant to Improve Rapeseed Oil Quality during Accelerated Shelf Life. Materials.

[126] Schar A., Liphardt S., Nystrom L. (2017). Enzymatic synthesis of steryl hydroxycinnamates and their antioxidant activity. Eur. J. Lipid Sci. Technol..

[127] Rabiej-Kozioł D., Krzemiński M.P., Szydłowska-Czerniak A. (2020). Synthesis of Steryl Hydroxycinnamates to Enhance Antioxidant Activity of Rapeseed Oil and Emulsions. Materials.

[128] Tan Z., Shahidi F. (2012). A novel chemoenzymatic synthesis of phytosteryl caffeates and assessment of their antioxidant activity. Food Chem..

[129] Wang S., Ye K., Shu T., Tang X., Wang X.J., Liu S. (2019). Enhancement of Galloylation Efficacy of Stigmasterol and β-Sitosterol Followed by Evaluation of Cholesterol-Reducing Activity. J. Agric. Food Chem..

[130] Wang H., Jia C., Xia X., Karangwa E., Zhang X. (2018). Enzymatic synthesis of phytosteryl lipoate and its antioxidant properties. Food Chem..

[131] Ras R.T., Hiemstra H., Lin Y., Vermeer M.A., Duchateau G.S., Trautwein E.A. (2013). Consumption of plant sterol-enriched foods and effects on plasma plant sterol concentrations-a meta-analysis of randomized controlled studies. Atherosclerosis.

[132] Piironen V., Lampi A.-M., Dutta P.C. (2004). 1. Occurrence and Levels of Phytosterols in Foods. Phytosterols as Functional Food Components and Nutraceuticals.

[133] Awad A.B., Chan K.C., Downie A.C., Fink C.S. (2000). Peanuts as a source of β-Sitosterol, a sterol with anticancer properties. Nutr. Cancer.

[134] Duester K.C. (2001). Avocado fruit is a rich source of beta-sitosterol. J. Am. Diet. Assoc..

[135] D’Evoli L., Lucarini M., Gabrielli P., Aguzzi A., Lombardi-Boccia G. (2015). Nutritional Value of Italian Pistachios from Bronte (*Pistacia vera*, L.), Their Nutrients, Bioactive Compounds and Antioxidant Activity. Food Nutr. Sci..

[136] Chong W.T., Lee Y.Y., Tang T.K., Phuah E.T., Lee Y., Tang T.K., Phuah E.T., Lai O.M. (2022). Minor Components in Edible Oil. Recent Advances in Edible Fats and Oils Technology.

[137] Behrman E.J., Gopalan V. (2005). Cholesterol and plants. J. Chem. Educ..

[138] Brzeska M., Szymczyk K., Szterk A. (2016). Current Knowledge about Oxysterols: A Review. J. Food Sci..

[139] Turck D., Castenmiller J., De Henauw S., Hirsch-Ernst K.I., Kearney J., Maciuk A., Mangelsdorf I., McArdle H.J., Naska A., EFSA NDA Panel (EFSA Panel on Nutrition, Novel Foods and Food Allergens) (2020). Scientific Opinion on the safety of the extension of use of plant sterol esters as a novel food pursuant to Regulation (EU) 2015/2283. EFSA J..

[140] Garcia-Llatas G., Mercatante D., López-García G., Rodriguez-Estrada M.T. (2021). Oxysterols—How much do we know about food occurrence, dietary intake and absorption?. Curr. Opin. Food Sci..

[141] Menéndez-Carreño M., Knol D., Janssen H.G. (2016). Development and validation of methodologies for the quantification of phytosterols and phytosterol oxidation products in cooked and baked food products. J. Chromatogr. A.

[142] Ostlund R.E., McGill J.B., Zeng C.M., Covey D.F., Stearns J., Stenson W.F., Spilburg C.A. (2002). Gastrointestinal absorption and plasma kinetics of soy Delta(5)-phytosterols and phytostanols in humans. Am. J. Physiol. Endocrinol. Metab..

[143] Kritchevsky D., Werthessen N.T., Shapiro I.L., Nair P.P., Turner D.A. (1965). Transfer of radioactivity of cholesterol-7-a3 H to fatty acids in tissue lipids in vivo. Nature.

[144] Duchateau G., Cochrane B., Windebank S., Herudzinska J., Sanghera D., Burian A., Müller M., Zeitlinger M., Lappin G. (2012). Absolute oral bioavailability and metabolic turnover of β-sitosterol in healthy subjects. Drug Metab. Dispos..

[145] Yoshida H., Tada H., Ito K., Kishimoto Y., Yanai H., Okamura T., Ikewaki K., Inagaki K., Shoji T., Bujo H. (2020). Reference Intervals of Serum Non-Cholesterol Sterols by Gender in Healthy Japanese Individuals. J. Atheroscler. Thromb..

[146] Fassbender K., Lutjohann D., Dik M.G., Bremmer M., Konig J., Walter S., Liu Y., Letiembre M., von Bergmann K., Jonker C. (2008). Moderately elevated plant sterol levels are associated with reduced cardiovascular risk–the LASA study. Atherosclerosis.

[147] Nghiem-Rao T.H., Tunc I., Mavis A.M., Cao Y., Polzin E.M., Firary M.F., Wang X., Simpson P.M., Patel S.B. (2015). Kinetics of phytosterol metabolism in neonates receiving parenteral nutrition. Pediatr. Res..

[148] Baumgartner S., Mensink R.P., Husche C., Lütjohann D., Plat J. (2013). Effects of plant sterol- or stanol-enriched margarine on fasting plasma oxyphytosterol concentrations in healthy subjects. Atherosclerosis.

[149] Husche C., Weingärtner O., Pettersson H., Vanmierlo T., Böhm M., Laufs U., Lütjohann D. (2011). Validation of an isotope dilution gas chromatography-mass spectrometry method for analysis of 7-oxygenated campesterol and sitosterol in human serum. Chem. Phys. Lipids.

[150] Menéndez-Carreño M., Steenbergen H., Janssen H.G. (2012). Development and validation of a comprehensive two-dimensional gas chromatography-mass spectrometry method for the analysis of phytosterol oxidation products in human plasma. Anal. Bioanal. Chem..

[151] Kulig W., Cwiklik L., Jurkiewicz P., Rog T., Vattulainen I. (2016). Cholesterol oxidation products and their biological importance. Chem. Phys. Lipids.

[152] Schött H.F., Luister A., Husche C., Schäfers H.J., Böhm M., Plat J., Lütjohann D., Laufs U., Weingärtner O. (2014). The relationships of phytosterols and oxyphytosterols in plasma and aortic valve cusps in patients with severe aortic stenosis. Biochem. Biophys. Res. Commun..

[153] Baumgartner S., Lütjohann D., Husche C., Kerksiek A., Groen A.K., Mensink R.P., Plat J. (2022). Plasma oxyphytosterols most likely originate from hepatic oxidation and subsequent spill-over in the circulation. J. Steroid Biochem. Mol. Biol..

[154] Aringer L., Eneroth P., Nordström L. (1976). Side chain hydroxylation of cholesterol, campesterol and beta-sitosterol in rat liver mitochondria. J. Lipid Res..

[155] Subbiah M.T., Kuksis A. (1973). Differences in metabolism of cholesterol and sitosterol following intravenous injection in rats. Biochim. Biophys. Acta.

[156] Salen G., Ahrens E.H., Grundy S.M. (1970). Metabolism of beta-sitosterol in man. J. Clin. Investig..

[157] Boberg K.M., Einarsson K., Björkhem I. (1990). Apparent lack of conversion of sitosterol into C24-bile acids in humans. J. Lipid Res..

[158] Miettinen T.A., Vuoristo M., Nissinen M., Järvinen H.J., Gylling H. (2000). Serum, biliary, and fecal cholesterol and plant sterols in colectomized patients before and during consumption of stanol ester margarine. Am. J. Clin. Nutr..

[159] Sudhop T., Sahin Y., Lindenthal B., Hahn C., Lüers C., Berthold H.K., von Bergmann K. (2002). Comparison of the hepatic clearances of campesterol, sitosterol, and cholesterol in healthy subjects suggests that efflux transporters controlling intestinal sterol absorption also regulate biliary secretion. Gut.

[160] Czubayko F., Beumers B., Lammsfuss S., Lütjohann D., von Bergmann K. (1991). A simplified micro-method for quantification of fecal excretion of neutral and acidic sterols for outpatient studies in humans. J. Lipid Res..

[161] Schött H.F., Krautbauer S., Höring M., Liebisch G., Matysik S. (2018). A Validated, Fast Method for Quantification of Sterols and Gut Microbiome Derived 5α/β-Stanols in Human Feces by Isotope Dilution LC–High-Resolution MS. Anal. Chem..

[162] Sanders D.J., Minter H.J., Howes D., Hepburn P.A. (2000). The safety evaluation of phytosterol esters. Part 6. The comparative absorption and tissue distribution of phytosterols in the rat. Food Chem. Toxicol..

[163] Shafaati M., Marutle A., Pettersson H., Lövgren-Sandblom A., Olin M., Pikuleva I., Winblad B., Nordberg A., Björkhem I. (2011). Marked accumulation of 27-hydroxycholesterol in the brains of Alzheimer’s patients with the Swedish APP 670/671 mutation. J. Lipid Res..

[164] Tonello A., Poli G. (2006). Serum phytosterols not only from dietary intake. Br. J. Nutr..

[165] Bhattacharyya A.K., Connor W.E., Lin D.S. (1983). The origin of plant sterols in the skin surface lipids in humans: From diet to plasma to skin. J. Investig. Dermatol..

[166] Heverin M., Bogdanovic N., Lütjohann D., Bayer T., Pikuleva I., Bretillon L., Diczfalusy U., Winblad B., Björkhem I. (2004). Changes in the levels of cerebral and extracerebral sterols in the brain of patients with Alzheimer’s disease. J. Lipid Res..

[167] Vanmierlo T., Bogie J.F., Mailleux J., Vanmol J., Lütjohann D., Mulder M., Hendriks J.J. (2015). Plant sterols: Friend or foe in CNS disorders?. Prog. Lipid Res..

[168] Teupser D., Baber R., Ceglarek U., Scholz M., Illig T., Gieger C., Holdt L.M., Leichtle A., Greiser K.H., Huster D. (2010). Genetic regulation of serum phytosterol levels and risk of coronary artery disease. Circ. Cardiovasc. Genet..

[169] Tada H., Nohara A., Inazu A., Sakuma N., Mabuchi H., Kawashiri M.A. (2018). Sitosterolemia, Hypercholesterolemia, and Coronary Artery Disease. J. Atheroscler. Thromb..

[170] Krawczyk M., Lütjohann D., Schirin-Sokhan R., Villarroel L., Nervi F., Pimentel F., Lammert F., Miquel J.F. (2012). Phytosterol and cholesterol precursor levels indicate increased cholesterol excretion and biosynthesis in gallstone disease. Hepatology.

[171] Field F.J., Mathur S.N. (1983). Beta-sitosterol: Esterification by intestinal acylcoenzyme A: Cholesterol acyltransferase (ACAT) and its effect on cholesterol esterification. J. Lipid Res..

[172] Temel R.E., Gebre A.K., Parks J.S., Rudel L.L. (2003). Compared with Acyl-CoA:cholesterol O-acyltransferase (ACAT) 1 and lecithin:cholesterol acyltransferase, ACAT2 displays the greatest capacity to differentiate cholesterol from sitosterol. J. Biol. Chem..

[173] Tachibana S., Hirano M., Hirata T., Matsuo M., Ikeda I., Ueda K., Sato R. (2007). Cholesterol and plant sterol efflux from cultured intestinal epithelial cells is mediated by ATP-binding cassette transporters. Biosci. Biotechnol. Biochem..

[174] Field F.J., Born E., Mathur S.N. (2004). LXR/RXR ligand activation enhances basolateral efflux of beta-sitosterol in CaCo-2 cells. J. Lipid Res..

[175] van der Wulp M.Y., Verkade H.J., Groen A.K. (2013). Regulation of cholesterol homeostasis. Mol. Cell. Endocrinol..

[176] Kanuri B., Fong V., Patel S.B., Ntambi J. (2020). 24 Role of Xenosterols in Health and Disease. Lipid Signaling and Metabolism.

[177] Taha D.A., Wasan E.K., Wasan K.M., Gershkovich P. (2015). Lipid-lowering Activity of Natural and Semi-Synthetic Sterols and Stanols. J. Pharm. Pharm. Sci..

[178] JECFA (Joint FAO/WHO Expert Committee on Food Additives) (2009). Phytosterols, phytostanols and their esters. Toxicological Evaluation of Certain Food Additives.

[179] SCF (Scientific Committee on Food) Opinion of the Scientific Committee on Food on Applications for Approval of a Variety of Plant Sterol-Enriched Foods, (Expressed on 5 March 2003); Scientific Committee on Food (SCF), European Commission, Health & Consumer Protection Directorate-General, Directorate C—Scientific Opinions, C2—Management of Scientific Committees, Scientific Co-Operation and Networks, Brussels, Belgium, 2003 (SCF/CS/NF/DOS/15 ADD 2 Final). https://mobil.bfr.bund.de/cm/343/phytosterol_fazer_teriaki_puottu_march_2003.pdf.

[180] Noakes M., Clifton P., Ntanios F., Shrapnel W., Record I., McInerney J. (2002). An increase in dietary carotenoids when consuming plant sterols or stanols is effective in maintaining plasma carotenoid concentrations. Am. J. Clin. Nutr..

[181] Feng S., Wang L., Shao P., Sun P., Yang C.S. (2022). A review on chemical and physical modifications of phytosterols and their influence on bioavailability and safety. Crit. Rev. Food. Sci. Nutr..

[182] Fuhrmann A., Weingärtner O., Meyer S., Cremers B., Seiler-Mußler S., Schött H.F., Kerksiek A., Friedrichs S., Ulbricht U., Zawada A.M. (2018). Plasma levels of the oxyphytosterol 7α-hydroxycampesterol are associated with cardiovascular events. Atherosclerosis.

[183] Takayasu B.S., Martins I.R., Garnique A.M.B., Miyamoto S., Machado-Santelli G.M., Uemi M., Onuki J. (2020). Biological effects of an oxyphytosterol generated by β-Sitosterol ozonization. Arch. Biochem. Biophys..

[184] Aguilar F., Crebelli R., Dusemund B., Galtier P., Gilbert J., Gott D.M., Gundert-Remy U., König J., Lambré C., Leblanc J.-C. (2012). Scientific Opinion on the safety of stigmasterol-rich plant sterols as food additive. EFSA J..

[185] Phillips K.M., Ruggio D.M., Ashraf-Khorassani M. (2005). Phytosterol composition of nuts and seeds commonly consumed in the United States. J. Agric. Food Chem..

[186] Muenger L.H., Jutzi S., Lampi A.-M., Nyström L. (2015). Comparison of Enzymatic Hydrolysis and Acid Hydrolysis of Sterol Glycosides from Foods Rich in Delta(7)-Sterols. Lipids.

[187] Garcia-Llatas G., Alegría A., Barberá R., Cilla A. (2021). Current methodologies for phytosterol analysis in foods. Microchem. J..

[188] Toivo J., Phillips K., Lampi A.M., Piironen V. (2001). Determination of sterols in foods: Recovery of free, esterified, and glycosidic sterols. J. Food Compos. Anal..

[189] Heupel R.C., Nes W.D., Parish E.J. (1989). 1-Isolation and Primary Characterization of Sterols. Analysis of Sterols and Other Biologically Significant Sterols.

[190] Lampi A.M., Juntunen L., Toivo J., Piironen V. (2002). Determination of thermo-oxidation products of plant sterols. J. Chromatogr. B Analyt. Technol. Biomed. Life Sci..

[191] Zhang X., Julien-David D., Miesch M., Geoffroy P., Raul F., Roussi S., Aoudé-Werner D., Marchioni E. (2005). Identification and quantitative analysis of beta-sitosterol oxides in vegetable oils by capillary gas chromatography-mass spectrometry. Steroids.

[192] Mendiara I., Domeño C., Nerín C. (2012). Development of a fast sample treatment for the analysis of free and bonded sterols in human serum by LC-MS. J. Sep. Sci..

[193] Yasui K., Pollet B.G. (2018). Acoustic cavitation. Acoustic Cavitation and Bubble Dynamics, (SpringerBriefs in Molecular Science: Ultrasound and Sonochemistry).

[194] Cravotto G., Cintas P. (2007). Forcing and controlling chemical reactions with ultrasound. Angew. Chem. Int. Ed. Engl..

[195] Luche J.-L., Einhorn C., Einhorn J., Sinisterra-Gago J.V. (1990). Organic sonochenistry: A new interpretation and its consequences. Tetrahedron Lett..

[196] Folch J., Lees M., Sloane Stanley G.H. (1957). A simple method for the isolation and purification of total lipides from animal tissues. J. Biol. Chem..

[197] Bligh E.G., Dyer W.J. (1959). A rapid method of total lipid extraction and purification. Can. J. Biochem. Physiol..

[198] Ms U., Ferdosh S., Akanda J.H., Ghafoor K., Rukshana A.H., Ali E., Kamaruzzaman B.Y., Fauzi M.B., Hadijah S., Shaarani S. (2018). Techniques for the extraction of phytosterols and their benefits in human health: A review. Sep. Sci. Technol..

[199] Luo H.F., Li Q., Yu S., Badger T.M., Fang N. (2005). Cytotoxic hydroxylated triterpene alcohol ferulates from rice bran. J. Nat. Prod..

[200] McDonald J.G., Thompson B.M., McCrum E.C., Russell D.W. (2007). Extraction and analysis of sterols in biological matrices by high performance liquid chromatography electrospray ionization mass spectrometry. Methods Enzymol..

[201] Farines M., Cocallemen S., Soulier J. (1988). Triterpene alcohols, 4-methylsterols, and 4-desmethylsterols of eggplant seed oil: A new phytosterol. Lipids.

[202] Azadmard-Damirchi S., Nemati M., Hesari J., Ansarin M., Fathi-Achachlouei B. (2010). Rapid separating and enrichment of 4,4′-dimethylsterols of vegetable oils by solid-phase extraction. J. Am. Oil Chem. Soc..

[203] Kornfeldt A., Croon L.-B. (1981). 4-Demethyl, 4-monomethyl, and 4,4-dimethylsterols in some vegetable oils. Lipids.

[204] Kamal-Eldin A., Appelqvist L.-Å., Yousif G., Iskander G.M. (1992). Seed lipids of *Sesamum indicum* and related wild species in Sudan. The sterols. J. Sci. Food Agric..

[205] Hartmann M.-A., Benveniste P. (1987). Plant membrane sterols: Isolation, identification, and biosynthesis. Meth. Enzymol..

[206] Azadmard-Damirchi S., Dutta P. (2006). Novel solid-phase extraction method to separate 4-desmethyl-, 4-monomethyl-, and 4,4′-dimethylsterols in vegetable oils. J. Chromatogr. A.

[207] Phillips K.M., Ruggio D.M., Toivo J.I., Swank M.A., Simpkins A.H. (2002). Free and esterified sterol composition of edible oils and fats. J. Food Comp. Anal..

[208] Phillips K.M., Ruggio D.M., Bailey J.A. (1999). Precise quantitative determination of phytosterols, stanols, and cholesterol metabolites in human serum by capillary gas-liquid chromatography. J. Chromatogr. B.

[209] Toivo J., Lampi A.-M., Aalto S., Piironen V. (2000). Factors affecting sample preparation in the gas chromatographic determination of plant sterols in whole wheat flour. Food Chem..

[210] Esche R., Barnsteiner A., Scholz B., Engel K.H. (2012). Simultaneous Analysis of free phytosterol/phytostanol and intact phytosteryl/phytostanyl fatty acid and phenolic acid esters in cereals. J. Agric. Food Chem..

[211] Xu B., You S., Zhou L., Kang H., Luo D., Ma H., Han S. (2020). Simultaneous Determination of Free Phytosterols and Tocopherols in Vegetable Oils by an Improved SPE–GC–FID Method. Food Anal. Methods.

[212] Lechner M., Reiter B., Lorbeer E. (1999). Determination of tocopherols and sterols in vegetable oils by solid-phase extraction and subsequent capillary gas chromatographic analysis. J. Chromatogr. A.

[213] Rose-Sallin C., Huggett A., Bosset J.O., Tabacchi R., Fay L.B. (1995). Quantification of cholesterol oxidation products in milk powders using [2H7] cholesterol to monitor cholesterol autoxidation artifacts. J. Agric. Food Chem..

[214] Griffiths W.J., Abdel-Khalik J., Crick P.J., Yutuc E., Wang Y. (2016). New methods for analysis of oxysterols and related compounds by LC-MS. J. Steroid Biochem. Mol. Biol..

[215] Nystrom L., Schar A., Lampi A.M. (2012). Steryl glycosides and acylated steryl glycosides in plant foods reflect unique sterol patterns. Eur. J. Lipid Sci. Technol..

[216] Balme S., Gülaçar F.O. (2012). Rapid screening of phytosterols in orange juice by solid-phase microextraction on polyacrylate fibre derivatisation and gas chromatographic–mass spectrometric. Food Chem..

[217] Domeño C., Ruiz B., Nerín C. (2005). Determination of sterols in biological samples by SPME with on-fiber derivatization and GC/FID. Anal. Bioanal. Chem..

[218] Halket J.M., Zaikin V.G. (2003). Derivatization in Mass Spectrometry—1. Silylation. Eur. J. Mass Spectrom..

[219] Harkin C., Smith K.W., Cruickshank F.L., Logan Mackay C., Flinders B., Heeren R.M.A., Moore T., Brockbank S., Cobice D.F. (2022). On-tissue chemical derivatization in mass spectrometry imaging. Mass Spectrom. Rev..

[220] Moldoveanu S.C., David V., Kusch P. (2019). Derivatization Methods in GC and GC/MS. Gas Chromatography—Derivatization, Sample Preparation, Application.

[221] Li T., Yin Y., Zhou Z., Qiu J., Liu W., Zhang X., He K., Cai Y., Zhu Z.-J. (2021). Ion mobility-based sterolomics reveals spatially and temporally distinctive sterol lipids in the mouse brain. Nat. Commun..

[222] Qi W., Wang Y., Cao Y., Cao Y., Guan Q., Sun T., Zhang L., Guo Y. (2020). Simultaneous Analysis of Fatty Alcohols, Fatty Aldehydes, and Sterols in Thyroid Tissues by Electrospray Ionization-Ion Mobility-Mass Spectrometry Based on Charge Derivatization. Anal. Chem..

[223] (2014). Determination of Individual and Total Sterols Contents—Gas Chromatographic Method—Part 1: Animal and Vegetable Fats and Oils, Corrected Version 2015-05-15.

[224] Řimnáčová L., Hušek P., Šimek P. (2014). A new method for immediate derivatization of hydroxyl groups by fluoroalkyl chloroformates and its application for the determination of sterols and tocopherols in human serum and amniotic fluid by gas chromatography-mass spectrometry. J. Chromatogr. A.

[225] Scholz B., Wocheslander S., Lander V., Engel K.-H. (2015). On-line liquid chromatography–gas chromatography: A novel approach for the analysis of phytosterol oxidation products in enriched foods. J. Chromatogr. A.

[226] Hailat I., Helleur R.J. (2014). Direct analysis of sterols by derivatization matrix-assisted laser desorption/ionization time-of-flight mass spectrometry and tandem mass spectrometry. Rapid Commun. Mass Spectrom..

[227] Boenzi S., Deodato F., Taurisano R., Martinelli D., Verrigni D., Carrozzo R., Bertini E., Pastore A., Dionisi-Vici C., Johnson D.W. (2014). A New Simple and Rapid LC–ESI-MS/MS Method for Quantification of Plasma Oxysterols as Dimethylaminobutyrate Esters. Its Successful Use for the Diagnosis of Niemann–Pick Type C Disease. Clin. Chim. Acta.

[228] Roberg-Larsen H., Lund K., Vehus T., Solberg N., Vesterdal C., Misaghian D., Olsen P.A., Krauss S., Wilson S.R., Lundane E. (2014). Highly Automated Nano-LC/MS-Based Approach for Thousand Cell-Scale Quantification of Side Chain-Hydroxylated Oxysterols. J. Lipid Res..

[229] Honda A., Yamashita K., Hara T., Ikegami T., Miyazaki T., Shirai M., Xu G., Numazawa M., Matsuzaki Y. (2009). Highly sensitive quantification of key regulatory oxysterols in biological samples by LC-ESI-MS/MS. J. Lipid Res..

[230] Nzekoue F.K., Caprioli G., Ricciutelli M., Cortese M., Alesi A., Vittori S., Sagratini G. (2020). Development of an innovative phytosterol derivatization method to improve the HPLC-DAD analysis and the ESI-MS detection of plant sterols/stanols. Food Res. Int..

[231] Griffiths W.J., Hearn T., Crick P.J., Abdel-Khalik J., Dickson A., Yutuc E., Wang Y. (2017). Charge-tagging liquid chromatography-mass spectrometry methodology targeting oxysterol diastereoisomers. Chem. Phys. Lipids.

[232] Crick P.J., Bentley T.W., Abdel-Khalik J., Matthews I., Clayton P.T., Morris A.A., Bigger B.W., Zerbinati C., Tritapepe L., Iuliano L. (2015). Quantitative charge-tags for sterol and oxysterol analysis. Clin. Chem..

[233] Adhikari S., Xia Y. (2017). Thiyl Radical-Based Charge Tagging Enables Sterol Quantitation via Mass Spectrometry. Anal. Chem..

[234] Xie X., Zhao J., Lin M., Zhang J.L., Xia Y. (2020). Profiling of Cholesteryl Esters by Coupling Charge-Tagging Paternò-Büchi Reaction and Liquid Chromatography-Mass Spectrometry. Anal. Chem..

[235] Marshall D.L., Criscuolo A., Young R.S.E., Poad B.L.J., Zeller M., Reid G.E., Mitchell T.W., Blanksby S.J. (2019). Mapping Unsaturation in Human Plasma Lipids by Data-Independent Ozone-Induced Dissociation. J. Am. Soc. Mass. Spectrom..

[236] Lognay G., Severin M., Boenke A., Wagstaffe P.J. (1992). Edible fats and oils reference materials for sterols analysis particular attention to cholesterol. Part 1. Investigation of some analytical aspects by experienced laboratorios. Analyst.

[237] Laakso P. (2005). Analysis of sterols from various food matrices. Eur. J. Lipid Sci. Technol..

[238] Matysik S., Klünemann H.H., Schmitz G. (2012). Gas chromatography-tandem mass spectrometry method for the simultaneous determination of oxysterols, plant sterols, and cholesterol precursors. Clin. Chem..

[239] Schött H.-F., Lütjohann D. (2015). Validation of an isotope dilution gas chromatography–mass spectrometry method for combined analysis of oxysterols and oxyphytosterols in serum samples. Steroids.

[240] Ito J., Shimizu N., Kato S., Ogura Y., Nakagawa K. (2020). Direct Separation of the Diastereomers of Cholesterol Ester Hydroperoxide Using LC-MS/MS to Evaluate Enzymatic Lipid Oxidation. Symmetry.

[241] Sosińska E., Przybylski R., Hazendonk P., Zhao Y.Y., Curtis J.M. (2013). Characterisation of non-polar dimers formed during thermo-oxidative degradation of β-sitosterol. Food Chem..

[242] Tomono S., Miyoshi N., Ito M., Higashi T., Ohshima H. (2011). A highly sensitive LC-ESI-MS/MS method for the quantification of cholesterol ozonolysis products secosterol-A and secosterol-B after derivatization with 2-hydrazino-1-methylpyridine. J. Chromatogr. B Analyt. Technol. Biomed. Life Sci..

[243] Hu Y., Yang G., Huang W., Lai S., Ren Y., Huang B., Zhang L., Li P., Lu B. (2015). Development and validation of a gas chromatography-mass spectrometry method for determination of sterol oxidation products in edible oils. RSC Adv..

[244] Islam A., Jeong B.-G., Kerr W.L., Chun J. (2021). Validation of phytosterol analysis by alkaline hydrolysis and trimethylsilyl derivatization coupled with gas chromatography for rice products. J. Cereal Sci..

[245] Lin Y., Koppenol W.P., Knol D., Vermeer M.A., Hiemstra H., Friedrichs S., Lütjohann D., Trautwein E.A. (2019). Serum Concentration of Plant Sterol Oxidation Products (POP) Compared to Cholesterol Oxidation Products (COP) after Intake of Oxidized Plant Sterols: A Randomised, Placebo-Controlled, Double-Blind Dose–Response Pilot Study. Nutrients.

[246] Menéndez-Carreño M., García-Herreros C., Astiasarán I., Ansorena D. (2008). Validation of a gas chromatography-mass spectrometry method for the analysis of sterol oxidation products in serum. J. Chromatogr. B Analyt. Technol. Biomed. Life Sci..

[247] Oliveira L., Freire C.S.R., Silvestre A.J.D., Cordeiro N., Torres I.C., Evtuguin D. (2005). Steryl Glucosides from Banana Plant Musa Acuminata Colla Var Cavendish. Ind. Crops Prod..

[248] Prinsen P., Gutiérrez A., Faulds C.B., del Río J.C. (2014). Comprehensive study of valuable lipophilic phytochemicals in wheat bran. J. Agric. Food Chem..

[249] Poole C.F., Tranchida P.Q. (2020). Chapter 3 Conventional Gas Chromatography: Basic Principles and Instrumental Aspects. Advanced Gas Chromatography in Food Analysis.

[250] Harvey D., Vouros P. (2020). Mass Spectrometric Fragmentation of Trimethylsilyl and Related Alkylsilyl Derivatives. Mass Spec. Rev..

[251] González-Larena M., García-Llatas G., Vidal M.C., Sánchez-Siles L.M., Barberá R., Lagarda M.J. (2011). Stability of plant sterols in ingredients used in functional foods. J. Agric. Food Chem..

[252] Rudziñska M., Korczak J., Wasowicz E. (2005). Changes in phytosterols and their oxidation products during frying of French fries in rapeseed oil. Pol. J. Food Nutr. Sci..

[253] Schött H.F., Husche C., Friedrichs S., Miller C.M., McCarthy F.O., Laufs U., Plat J., Weingärtner O., Lütjohann D. (2015). 7β-Hydroxysitosterol crosses the blood-brain barrier more favored than its substrate sitosterol in ApoE-/-mice. Steroids.

[254] Rahier A., Benveniste P., Nes W.D., Parish E.J. (1989). Mass spectral identification of phytosterols. Analysis of Sterols and Other Biologically Significant Steroids.

[255] Tan S., Niu Y., Liu L., Su A., Hu C., Meng Y. (2019). Development of a GC-MS/SIM method for the determination of phytosteryl esters. Food Chem..

[256] Xu B.C., Zhang L.X., Wang H., Luo D.L., Li P.W. (2014). Characterization and authentication of four important edible oils using free phytosterol profiles established by GC-GC-TOF/MS. Anal. Methods.

[257] Tranchida P.Q., Salivo S., Franchina F.A., Bonaccorsi I., Dugo P., Mondello L. (2013). Qualitative and quantitative analysis of the unsaponifiable fraction of vegetable oils by using comprehensive 2D GC with dual MS/FID detection. Anal. Bioanal. Chem..

[258] Julien-David D., Zhao M., Geoffroy P., Miesch M., Raul F., Aoude-Werner D., Ennahar S., Marchioni E. (2014). Analysis of sitosteryl oleate esters in phytosterols esters enriched foods by HPLC-ESI-MS^2^. Steroids.

[259] Millán L., Sampedro M.C., Sánchez A., Delporte C., Van Antwerpen P., Goicolea M.A., Barrio R.J. (2016). Liquid chromatography-quadrupole time of flight tandem mass spectrometry-based targeted metabolomic study for varietal discrimination of grapes according to plant sterols content. J. Chromatogr. A.

[260] Caboni M.F., Iafelice G., Pelillo M., Marconi E. (2005). Analysis of fatty acid steryl esters in tetraploid and hexaploid wheats: Identification and comparison between chromatographic methods. J. Agric. Food Chem..

[261] Lembcke J., Ceglarek U., Fiedler G.M., Baumann S., Leichtle A., Thiery J. (2005). Rapid quantification of free and esterified phytosterols in human serum using APPI-LC-MS/MS. J. Lipid Res..

[262] Scholz B., Barnsteiner A., Feist K., Schmid W., Engel K.H. (2014). Analysis of phytostanyl fatty acid esters in enriched foods via UHPLC-APCI-MS. J. Agric. Food Chem..

[263] Ishida N. (2014). A method for simultaneous analysis of phytosterols and phytosterol esters in tobacco leaves using non aqueous reversed phase chromatography and atmospheric pressure chemical ionization mass spectrometry detector. J. Chromatogr. A.

[264] Mezine I., Zhang H., Macku C., Lijana R. (2003). Analysis of plant sterol and stanol esters in cholesterol-lowering spreads and beverages using high-performance liquid chromatography-atmospheric pressure chemical ionization-mass spectroscopy. J. Agric. Food Chem..

[265] Scholz B., Menzel N., Lander V., Engel K.H. (2016). An approach based on ultrahigh performance liquid chromatography-atmospheric pressure chemical ionization-mass spectrometry allowing the quantification of both individual phytosteryl and phytostanyl fatty acid esters in complex mixtures. J. Chromatogr. A.

[266] Broughton R., Ruíz-Lopez N., Hassall K.L., Martínez-Force E., Garcés R., Salas J.J., Beaudoin F. (2018). New insights in the composition of wax and sterol esters in common and mutant sunflower oils revealed by ESI-MS/MS. Food Chem..

[267] Wewer V., Dombrink I., vom Dorp K., Dörmann P. (2011). Quantification of sterol lipids in plants by quadrupole time-of-flight mass spectrometry. J. Lipid Res..

[268] Hailat I., Helleur R. (2014). Identification of fatty acid steryl esters in margarine and corn using direct flow injection ESI-MSn ion trap-mass spectrometry. Int. J. Mass Spectrom..

[269] Yang K., Han X. (2011). Accurate quantification of lipid species by electrospray ionization mass spectrometry—Meet a key challenge in lipidomics. Metabolites.

[270] Broughton R., Beaudoin F. (2021). Analysis of Free and Esterified Sterol Content and Composition in Seeds Using GC and ESI-MS/MS. Methods Mol. Biol..

[271] Jiang K., Gachumi G., Poudel A., Shurmer B., Bashi Z., El-Aneed A. (2019). The Establishment of Tandem Mass Spectrometric Fingerprints of Phytosterols and Tocopherols and the Development of Targeted Profiling Strategies in Vegetable Oils. J. Am. Soc. Mass Spectrom..

[272] Gorassini A., Verardo G., Fregolent S.C., Bortolomeazzi R. (2017). Rapid determination of cholesterol oxidation products in milk powder-based products by reversed phase SPE and HPLC-APCI-MS/MS. Food Chem..

[273] Poudel A., Gachumi G., Purves R., Badea I., El-Aneed A. (2022). Determination of phytosterol oxidation products in pharmaceutical liposomal formulations and plant vegetable oil extracts using novel fast liquid chromatography—Tandem mass spectrometric methods. Anal. Chim. Acta.

[274] Rossmann B., Thurner K., Luf W. (2007). MS–MS Fragmentation Patterns of Cholesterol Oxidation Products. Monatsh. Chem..

[275] Decloedt A.I., Van Landschoot A., Vanhaecke L. (2016). Fractional factorial design-based optimisation and application of an extraction and UPLC-MS/MS detection method for the quantification of phytosterols in food, feed and beverages low in phytosterols. Anal. Bioanal. Chem..

[276] Millán L., Sampedro M.C., Sanchez A., Goicolea M.A., Barrio R.J. (2015). Determination of phytosterols in oenological matrices by liquid chromatography-atmospheric pressure chemical ionization and ion-trap mass spectrometry. J. Food Compos. Anal..

[277] Donato P., Micalizzi G., Oteri M., Rigano F., Sciarrone D., Dugo P., Mondello L. (2018). Comprehensive lipid profiling in the Mediterranean mussel (*Mytilus galloprovincialis*) using hyphenated and multidimensional chromatography techniques coupled to mass spectrometry detection. Anal. Bioanal. Chem..

[278] Murphy R.C. (2015). CHAPTER 7 Steroids. Tandem Mass Spectrometry of Lipids: Molecular Analysis of Complex Lipids.

[279] Nes W.D., Robert A., Norton C., Mabry B. (1992). Carbon-13 NMR studies on sitosterol biosynthesized from [13C] mevalonates. Phytochemistry.

[280] Tsuda M., Schroepfer G.J. (1979). Nuclear magnetic resonance spectroscopy of sterols. ^13^C olefinic carbon shieldings in sterols and related cyclic compounds. J. Chem. Phys. Lipids.

[281] Emmons G.T., Wilson W.K., Shroepfer G.J. (1989). ^1^H and ^13^C NMR assignments for lanostan-3B-ol Derivatives: Revised assignments for lanosterol. Magn. Reason. Chem..

[282] Greca M.D., Monaco P., Previtera L. (1990). Stigmasterols from *Typha latifólia*. J. Nat. Prod..

[283] Murai T., Jin S., Itoh M., Horie Y., Higashi T., Ikegawa S. (2020). Analysis of steryl glucosides in rice bran-based fermented food by LC/ESI-MS/MS. Steroids.

[284] Wilson W.K., Sumpter R.M., Warren J.J., Rogers P.S., Ruan B., Schroepfer G.J. (1996). Analysis of unsaturated C27 sterols by nuclear magnetic resonance spectroscopy. J. Lipid Res..

[285] Van Hoed V., Zyaykina N., De Greyt W., Maes J., Verhe R., Demeestere K. (2008). Identification and occurrence of steryl glucosides in palm and soy biodiesel. J. Am. Oil Chem. Soc..

[286] Dixit P., Chand K., Khan M.P., Siddiqui J.A., Tewari D., Ngueguim F.T., Chattopadhyay N., Maurya R. (2012). Phytoceramides and acylated phytosterol glucosides from *Pterospermum acerifolium* Willd. seed coat and their osteogenic activity. Phytochemistry.

[287] McCarthy F.O., Chopra J., Ford A., Hogan S.A., Kerry J.P., O’Brien N.M., Ryan E., Maguire A.R. (2005). Synthesis, isolation and characterisation of beta-sitosterol and beta-sitosterol oxide derivatives. Org. Biomol. Chem..

[288] Shi T., Zhu M., Zhou X., Huo X., Long Y., Zeng X., Chen Y. (2019). ^1^H NMR combined with PLS for the rapid determination of squalene and sterols in vegetable oils. Food Chem..

[289] von Bonsdorff-Nikander A., Rantanen J., Christiansen L., Yliruusi J. (2003). Optimizing the crystal size and habit of beta-sitosterol in suspension. AAPS Pharm. Sci. Technol..

[290] Vu P.-L., Shin J.-A., Lim C.-H., Lee K.-T. (2004). Lipase-Catalyzed Production of Phytosteryl Esters and Their Crystallization Behavior in Corn Oil. Food Res. Int..

[291] Young N.W.G., Wassell P., Hasenhuettl G.L., Hartel R.W. (2019). Margarines and Spreads. Food Emulsifiers and Their Applications.

[292] Daels E., Foubert I., Guo Z., Thielemans W., Goderis B. (2020). Phase Behavior and Polymorphism of Saturated and Unsaturated Phytosterol Esters. Molecules.

[293] Bakrim S., Benkhaira N., Bourais I., Benali T., Lee L.-H., El Omari N., Sheikh R.A., Goh K.W., Ming L.C., Bouyahya A. (2022). Health Benefits and Pharmacological Properties of Stigmasterol. Antioxidants.

[294] Abeesh P., Guruvayoorappan C. (2020). Preparation and characterization of beta sitosterol encapsulated nanoliposomal formulation for improved delivery to cancer cells and evaluation of its anti-tumor activities against Daltons Lymphoma Ascites tumor models. J. Drug Deliv. Sci. Technol..

[295] Karim S., Akhter M.H., Burzangi A.S., Alkreathy H., Alharthy B., Kotta S., Md S., Rashid M.A., Afzal O., Altamimi A.S.A. (2022). Phytosterol-Loaded Surface Tailored Bioactive-Polymer Nanoparticles for Cancer Treatment: Optimization, in vitro Cell Viability, Antioxidant Activity, and Stability Studies. Gels.

[296] Kavithaa K., Paulpandi M., Ramya S., Ramesh M., Balachandar V., Ramasamy K., Narayanasamy A. (2021). Sitosterol-fabricated chitosan nanocomplex induces apoptotic cell death through mitochondrial dysfunction in lung cancer animal model: An enhanced synergetic drug delivery system for lung cancer therapy. New J. Chem..

[297] Nisha R., Kumar P., Gautam A.K., Bera H., Bhattacharya B., Parashar P., Saraf S.A., Saha S. (2021). Assessments of in vitro and in vivo antineoplastic potentials of β-sitosterol-loaded PEGylated niosomes against hepatocellular carcinoma. J. Liposome Res..

[298] Soleimanian Y., Goli S.A.H., Varshosaz J., Maestrelli F. (2018). Propolis wax nanostructured lipid carrier for delivery of β-sitosterol: Effect of formulation variables on physicochemical properties. Food Chem..

[299] Upadhyay K., Gupta N.K., Dixit V.K. (2012). Development and characterization of phyto-vesicles of β-sitosterol for the treatment of androgenetic alopecia. Arch. Dermatol. Res..

[300] Blanco-Vaca F., Cedó L., Julve J. (2019). Phytosterols in Cancer: From Molecular Mechanisms to Preventive and Therapeutic Potentials. Curr. Med. Chem..

[301] Jiang L., Zhao X., Xu J., Li C., Yu Y., Wang W., Zhu L. (2019). The Protective Effect of Dietary Phytosterols on Cancer Risk: A Systematic Meta-Analysis. J. Oncol..

[302] Ramprasath V.R., Awad A.B. (2015). Role of Phytosterols in Cancer Prevention and Treatment. J. AOAC Int..

[303] Woyengo T., Ramprasath V., Jones P. (2009). Anticancer effects of phytosterols. Eur. J. Clin. Nutr..

[304] Bao X., Zhang Y., Zhang H., Xia L. (2022). Molecular Mechanism of β-Sitosterol and its Derivatives in Tumor Progression. Front. Oncol..

[305] Nair P.P., Turjman N., Goodman G.T., Guidry C., Calkins B.M. (1984). Diet, nutrition intake, and metabolism in populations at high and low risk for colon cancer. Metabolism of neutral sterols. Am. J. Clin. Nutr..

[306] Huang J., Xu M., Fang Y.J., Lu M.S., Pan Z.Z., Huang W.Q., Chen Y.M., Zhang C.X. (2017). Association between phytosterol intake and colorectal cancer risk: A case-control study. Br. J. Nutr..

[307] Normén A.L., Brants H.A., Voorrips L.E., Andersson H.A., van den Brandt P.A., Goldbohm R.A. (2001). Plant sterol intakes and colorectal cancer risk in the Netherlands Cohort Study on Diet and Cancer. Am. J. Clin. Nutr..

[308] De Stefani E., Boffetta P., Ronco A.L., Brennan P., Deneo-Pellegrini H., Carzoglio J.C., Mendilaharsu M. (2000). Plant sterols and risk of stomach cancer: A case-control study in Uruguay. Nutr. Cancer.

[309] Mendilaharsu M., De Stefani E., Deneo-Pellegrini H., Carzoglio J., Ronco A. (1998). Phytosterols and risk of lung cancer: A case-control study in Uruguay. Lung Cancer.

[310] Ronco A., De Stefani E., Boffetta P., Deneo-Pellegrini H., Mendilaharsu M., Leborgne F. (1999). Vegetables, fruits, and related nutrients and risk of breast cancer: A case-control study in Uruguay. Nutr. Cancer.

[311] Torres-Sanchez L., Galvan-Portillo M., Wolff M.S., Lopez-Carrillo L. (2009). Dietary consumption of phytochemicals and breast cancer risk in Mexican women. Public Health Nutr..

[312] McCann S.E., Freudenheim J.L., Marshall J.R., Brasure J.R., Swanson M.K., Graham S. (2000). Diet in the epidemiology of endometrial cancer in western New York (United States). Cancer Causes Control..

[313] McCann S.E., Freudenheim J.L., Marshall J.R., Graham S. (2003). Risk of human ovarian cancer is related to dietary intake of selected nutrients, phytochemicals and food groups. J. Nutr..

[314] Bradford P.G., Awad A.B. (2010). Modulation of signal transduction in cancer cells by phytosterols. BioFactors.

[315] Villasenor I.M., Angelada J., Canlas A.P., Echegoyen D. (2002). Bioactivity Studies on β-Sitosterol and its Glucoside. Phytother. Res..

[316] Choi S., Kim K.W., Choi J.S., Han S.T., Park Y.I., Lee S.K., Kim J.S., Chung M.H. (2002). Angiogenic activity of beta-sitosterol in the ischaemia/reperfusion-damaged brain of Mongolian gerbil. Planta Med..

[317] Gogoi D., Pal A., Chattopadhyay P., Paul S., Deka R.C., Mukherjee A.K. (2018). First Report of Plant-Derived β-Sitosterol with Antithrombotic, in vivo Anticoagulant, and Thrombus-Preventing Activities in a Mouse Model. J. Nat. Prod..

[318] Singh K., Gupta R.S. (2016). Antifertility activity of β-sitosterol isolated from *Barleria prionitis* (L.) roots in male albino rats. Int. J. Pharm. Pharm. Sci..

[319] Phatangare N., Deshmukh K., Murade V., Naikwadi P., Hase D., Chavhan M., Velis H. (2017). Isolation and characterization of β-sitosterol from *Justicia gendarussa* burm. F.-An anti-inflammatory compound. Int. Pharmac. Phytochem. Res..

[320] Kim S.-J. (2017). The Ameliorative Effect of β-sitosterol on DNCB-induced Atopic Dermatitis in Mice. Biomed. Sci. Lett..

[321] Sudeep H.V., Venkatakrishna K., Amrutharaj B., Anitha, Shyamprasad K. (2019). A phytosterol-enriched saw palmetto supercritical CO_2_ extract ameliorates testosterone-induced benign prostatic hyperplasia by regulating the inflammatory and apoptotic proteins in a rat model. BMC Complement. Altern. Med..

[322] Ramalingam S., Packirisamy M., Karuppiah M., Vasu G., Gopalakrishnan R., Gothandam K., Thiruppathi M. (2020). Effect of β-sitosterol on glucose homeostasis by sensitization of insulin resistance via enhanced protein expression of PPRγ and glucose transporter 4 in high fat diet and streptozotocin-induced diabetic rats. Cytotechnology.

[323] Plat J., Hendrikx T., Bieghs V., Jeurissen M.L., Walenbergh S.M., van Gorp P.J., De Smet E., Konings M., Vreugdenhil A.C., Guichot Y.D. (2014). Protective role of plant sterol and stanol esters in liver inflammation: Insights from mice and humans. PLoS ONE.

[324] Fraile L., Crisci E., Córdoba L., Navarro M.A., Osada J., Montoya M. (2012). Immunomodulatory properties of beta-sitosterol in pig immune responses. Int. Immunopharmacol..

[325] Gumede N.M., Lembede B.W., Brooksbank R.L., Erlwanger K.H., Chivandi E. (2020). β-Sitosterol Shows Potential to Protect Against the Development of High-Fructose Diet-Induced Metabolic Dysfunction in Female Rats. J. Med. Food..

[326] Adebiyi O.E., Olayemi F.O., Olopade J.O., Tan N.-H. (2018). β-sitosterol enhances motor coordination, attenuates memory loss and demyelination in a vanadium-induced model of experimental neurotoxicity. Pathophysiology.

[327] Kurano M., Hasegawa K., Kunimi M., Hara M., Yatomi Y., Teramoto T., Tsukamoto K. (2018). Sitosterol prevents obesity-related chronic inflammation. Biochim. Biophys. Acta Mol. Cell. Biol. Lipids.

[328] Babu S., Krishnan M., Rajagopal P., Periyasamy V., Veeraraghavan V., Govindan R., Jayaraman S. (2020). Beta-sitosterol attenuates insulin resistance in adipose tissue via IRS-1/Akt mediated insulin signaling in high fat diet and sucrose induced type-2 diabetic rats. Eur. J. Pharmacol..

[329] Cui S., Jiang H., Chen L., Xu J., Sun W., Sun H., Xie Z., Xu Y., Yang F., Liu W. (2020). Design, synthesis and evaluation of wound healing activity for β-sitosterols derivatives as potent Na^+^/K^+^-ATPase inhibitors. Bioorg. Chem..

[330] Genser B., Silbernagel G., De Backer G., Bruckert E., Carmena R., Chapman M.J., Deanfield J., Descamps O.S., Rietzschel E.R., Dias K.C. (2012). Plant sterols and cardiovascular disease: A systematic review and meta-analysis. Eur. Heart J..

[331] Lottenberg A.M., Bombo R.P., Ilha A., Nunes V.S., Nakandakare E.R., Quintão E.C. (2012). Do clinical and experimental investigations support an antiatherogenic role for dietary phytosterols/stanols?. IUBMB Life.

[332] Ras R.T., Geleijnse J.M., Trautwein E.A. (2014). LDL-cholesterol-lowering effect of plant sterols and stanols across different dose ranges: A meta-analysis of randomised controlled studies. Br. J. Nutr..

[333] Ras R.T., van der Schouw Y.T., Trautwein E.A., Sioen I., Dalmeijer G.W., Zock P.L., Beulens J.W. (2015). Intake of phytosterols from natural sources and risk of cardiovascular disease in the European Prospective Investigation into Cancer and Nutrition-the Netherlands (EPIC-NL) population. Eur. J. Prev. Cardiol..

[334] Juste C., Gérard P. (2021). Cholesterol-to-Coprostanol Conversion by the Gut Microbiota: What We Know, Suspect, and Ignore. Microorganisms.

[335] Mattson F.H., Grundy S.M., Crouse J.R. (1982). Optimizing the effect of plant sterols on cholesterol absorption in man. Am. J. Clin. Nutr..

[336] Carden T.J., Hang J., Dussault P.H., Carr T.P. (2015). Dietary Plant Sterol Esters Must Be Hydrolyzed to Reduce Intestinal Cholesterol Absorption in Hamsters. J. Nutr..

[337] Lei L., Wang X., Huang W., Liu Y., Zheng F., Ma K.Y., Li Y.M., Wang L., Man S.W., Zhang C. (2015). Cholesterol side chain analogs but not its ether analogs possess cholesterol-lowering activity. Food Funct..

[338] Wang X., Huang W., Lei L., Liu Y., Ma K.Y., Li Y.M., Wang L., Huang Y., Chen Z.Y. (2015). Blockage of hydroxyl group partially abolishes the cholesterol-lowering activity of β-sitosterol. J. Funct. Foods..

[339] Chung D.W., Kim W.D., Noh S.K., Dong M.S. (2008). Effects of hydrophilic and lipophilic beta-sitosterol derivatives on cholesterol absorption and plasma cholesterol levels in rats. J. Agric. Food Chem..

[340] Dumolt J.H., Rideout T.C. (2017). The lipid-lowering effects and associated mechanisms of dietary phytosterol supplementation. Curr. Pharmaceut. Des..

[341] Amiot M.J., Knol D., Cardinault N., Nowicki M., Bott R., Antona C., Borel P., Bernard J.P., Duchateau G., Lairon D. (2011). Phytosterol ester processing in the small intestine: Impact on cholesterol availability for absorption and chylomicron cholesterol incorporation in healthy humans. J. Lipid Res..

[342] Batth R., Nicolle C., Cuciurean I.S., Simonsen H.T. (2020). Biosynthesis and Industrial Production of Androsteroids. Plants.

[343] Donova M.V. (2018). Microbiotechnologies for steroid production. Microbiol. Aust..

[344] Fernández-Cabezón L., Galán B., García J.L. (2018). New Insights on Steroid Biotechnology. Front. Microbiol..

[345] Szentirmai A. (1990). Microbial physiology of side chain degradation of sterols. J. Ind. Microbiol..

[346] Lo C.K., Pan C.P., Liu W.H. (2002). Production of testosterone from phytosterol using a single-step microbial transformation by a mutant of *Mycobacterium* sp. J. Ind. Microbiol. Biotechnol..

[347] Eisa M., El-Refai H., Amin M. (2016). Single step biotransformation of corn oil phytosterols to boldenone by a newly isolated *Pseudomonas aeruginosa*. Biotechnol. Rep..

[348] Commission Regulation (EU) No 739/2013 of 30 July 2013 Amending Annex II to Regulation (EC) No 1333/2008 of the European Parliament and of the Council as Regards the Use of Stigmasterol-Rich Plant Sterols as a Stabiliser in Ready-to-Freeze Alcoholic Cocktails, and the Annex to Commission Regulation (EU) No 231/2012 as Regards Specifications for Stigmasterol-Rich Plant Sterols Food Additive. http://eur-lex.europa.eu/legal-content/EN/TXT/PDF/?uri=CELEX:32013R0739&from=DE.

[349] Matheson A., Dalkas G., Clegg P.S., Euston S.R. (2018). Phytosterol-based edible oleogels: A novel way of replacing saturated fat in food. Nutr. Bull..

[350] Marangoni A.G., Garti N., Marangoni A.G., Garti N. (2011). 1. An Overview of the Past, Present, and Future of Organogels. Edible Oleogels: Structure and Health Implications.

[351] Bot A., Agterof W.G.M. (2006). Structuring of edible oils by mixtures of γ-oryzanol with β-sitosterol or related phytosterols. J. Am. Oil Chem. Soc..

[352] Chen X.W., Sun S., Yang G.L., Ma C.G. (2020). Engineering Phytosterol-Based Oleogels for Potential Application as Sustainable Petrolatum Replacement. RSC Adv..

[353] Li L., Wan W., Cheng W., Liu G., Han L. (2019). Oxidatively stable curcumin-loaded oleogels structured by β-sitosterol and lecithin: Physical characteristics and release behaviour in vitro. Int. J. Food Sci. Technol..

[354] Yang D.X., Chen X.W., Yang X.Q. (2018). Phytosterol-based oleogels self-assembled with monoglyceride for controlled volatile release. J. Sci. Food Agric..

[355] Inamdar Y.M. (2018). Preparation and Evaluation of Beta Sitosterol Nanogel: A Carrier Design for Targeted Drug Delivery System. Asian J. Pharm. Res. Dev..

[356] Zhang Y., Sun C., Wang C., Jankovic K.E., Dong Y. (2021). Lipids and Lipid Derivatives for RNA Delivery. Chem. Rev..

[357] Eygeris Y., Patel S., Jozic A., Sahay G. (2020). Deconvoluting Lipid Nanoparticle Structure for Messenger RNA Delivery. Nano Lett..

[358] Djekic L., Čalija B., Micov A., Tomic M., Stepanovic-Petrovic R. (2019). Topical hydrogels with escin β-sitosterol phytosome and escin: Formulation development and in vivo assessment of antihyperalgesic activity. Drug Dev. Res..

[359] Hiruta Y., Hattori Y., Kawano K., Obata Y., Maitani Y. (2006). Novel ultra-deformable vesicles entrapped with bleomycin and enhanced to penetrate rat skin. J. Control. Release..

[360] Munawar M., Khan M.S., Saeed M., Younas U., Farag M.R., Di Cerbo A., El-Shall N., Loschi A.R., Dhama K., Alagawany M. (2022). Phytosterol: Nutritional significance, health benefits, and its uses in poultry and livestock nutrition. Anim. Biotechnol..

[361] Wong A. (2014). Chemical and microbiological considerations of phytosterols and their relative efficacies in functional foods for the lowering of serum cholesterol levels in humans: A review. J. Funct. Foods.

[362] Zawistowski J., Smith J., Charter E. (2010). 17. Tangible health benefits of phytosterol functional foods. Functional Food Product Development.

[363] Nzekoue F.K., Khamitova G., Angeloni S., Sempere A.N., Tao J., Maggi F., Xiao J., Sagratini G., Vittori S., Caprioli G. (2020). Spent coffee grounds: A potential commercial source of phytosterols. Food Chem..

[364] Kalogeropoulos N., Chiou A., Pyriochou V., Peristeraki A., Karathanos V.T. (2012). Bioactive phytochemicals in industrial tomatoes and their processing byproducts. LWT Food Sci. Technol..

[365] Özdestan Ö., Erol T., Acar B. Phytosterols in rice bran and usage of rice bran in food industry. Proceedings of the 9th Baltic Conference on Food Science and Technology “Food for Consumer Well-Being” FOODBALT 2014 Conference Proceedings.

[366] Kreps F., Burčová Z., Jablonský M., Ház A., Frecer V., Kyselka J., Schmidt Š., Šurina I., Filip V. (2017). Bioresource of Antioxidant and Potential Medicinal Compounds from Waste Biomass of Spruce. ACS Sustain. Chem. Eng..

[367] Da Silva A.C., Jorge N. (2017). Bioactive compounds of oils extracted from fruits seeds obtained from agroindustrial waste. Eur. J. Lipid Sci. Technol..

[368] Attard T.M., McElroy C.R., Rezende C.A., Polikarpov I., Clark J.H., Hunt A.J. (2015). Sugarcane waste as a valuable source of lipophilic molecules. Ind. Crops Prod..

[369] Rodrigues P.F., Evtyugin D.D., Evtuguin D.V., Prates A. (2018). Extractives profiles in the production of sulphite dissolving pulp from *Eucalyptus globulus* wood. J. Wood Chem. Technol..

[370] Evtyugin D.D., Prates A., Domingues M.R., Casal S., Evtuguin D.V. (2023). New method for isolating β-sitosterol from bleaching effluent of sulphite pulp mill. Food Bioprod. Process..

[371] Fernandes P., Cabral J.M. (2007). Phytosterols: Applications and recovery methods. Bioresour. Technol..

[372] Venkatramesh M., Karunanandaa B., Sun B., Gunter C.A., Boddupalli S., Kishore G.M. (2003). Expression of a Streptomyces 3-hydroxysteroid oxidase gene in oilseeds for converting phytosterols to phytostanols. Phytochemistry.

[373] Schaeffer A., Bronner R., Benveniste P., Schaller H. (2001). The ratio of campesterol to sitosterol that modulates growth in *Arabidopsis* is controlled by STEROL METHYLTRANSFERASE 2;1. Plant J..

[374] Shimada T.L., Ueda T., Hara-Nishimura I. (2021). Excess sterol accumulation affects seed morphology and physiology in *Arabidopsis thaliana*. Plant Signal. Behav..

[375] Stymne S., Schaller H. (2010). Involvement of the phospholipid sterol acyltransferase1 in plant sterol homeostasis and leaf senescence. Plant Physiol..

[376] Takemoto T., Kusano G., Yamamoto N. (1966). Studies on the constituents of *Cimicifuga* spp. I. Constituents of *Cimicifuga acerina*. (1). Yakugaku Zasshi.

[377] Le Goff M., Le Ferrec E., Mayer C., Mimouni V., Lagadic-Gossmann D., Schoefs B., Ulmann L. (2019). Microalgal carotenoids and phytosterols regulate biochemical mechanisms involved in human health and disease prevention. Biochimie.

[378] Martinez-Nieto L., Moreno-Romero M.V. (1994). Sterolic composition of the unsaponifiable fraction of oil of avocado of several varieties. Grasas Aceites.

[379] Srividya N., Heidorn D.B., Lange B.M. (2014). Rapid purification of gram quantities of β-sitosterol from a commercial phytosterol mixture. BMC Res. Notes.

[380] Randhir A., Laird D.W., Maker G., Trengove R., Moheimani N.R. (2020). Microalgae: A potential sustainable commercial source of sterols. Algal Res..

[381] Tabas I., Vance D.E., Vance J.E. (2008). 3. Oxysterols in atherosclerosis. Biochemistry of Lipids, Lipoproteins and Membranes.

[382] Borel P., Desmarchelier C. (2018). Bioavailability of fat-soluble vitamins and phytochemicals in humans: Effects of genetic variation. Annu. Rev. Nutr..

[383] Morand C., Tomás-Barberán F.A. (2019). Contribution of plant food bioactives in promoting health effects of plant foods: Why look at interindividual variability?. Eur. J. Nutr..

[384] Moses T., Pollier J., Thevelein J.M., Goossens A. (2013). Bioengineering of plant (tri)terpenoids: From metabolic engineering of plants to synthetic biology in vivo and in vitro. New Phytol..

[385] Ferrer A., Altabella T., Arró M., Boronat A. (2017). Emerging roles for conjugated sterols in plants. Prog. Lipid Res..

[386] Jones P.J.H., Abumweis S.S. (2009). Phytosterols as functional food ingredients: Linkages to cardiovascular disease and cancer. Curr. Opin. Clin. Nutr. Metab. Care..

[387] Cuevas-Tena M., Bermúdez J.D., Silvestre R.L.Á., Alegría A., Lagarda M.J. (2019). Impact of colonic fermentation on sterols after the intake of a plant sterol-enriched beverage: A randomized, double-blind crossover trial. Clin. Nutr..

[388] Nzekoue F.K., Henle T., Caprioli G., Sagratini G., Hellwig M. (2020). Food Protein Sterylation: Chemical Reactions between Reactive Amino Acids and Sterol Oxidation Products under Food Processing Conditions. Foods.

[389] Lin X., Ma L., Racette S.B., Anderson Spearie C.L., Ostlund R.E. (2009). Phytosterol glycosides reduce cholesterol absorption in humans. Am. J. Physiol. Gastrointest. Liver. Physiol..

[390] AOAC International, Official Methods of Analysis Appendix K: Guidelines for Dietary Supplements and Botanicals; 2019; pp. 1–32. http://www.eoma.aoac.org/appk.pdf.

[391] Khamis M.M., Adamko D.J., El-Aneed A. (2021). Strategies and challenges in method development and validation for the absolute quantification of endogenous biomarker metabolites using liquid chromatography-tandem mass spectrometry. Mass Spectrom. Rev..

[392] Meesters R.J., Voswinkel S. (2018). Bioanalytical Method Development and Validation: From the USFDA 2001 to the USFDA 2018 Guidance for Industry. J. Appl. Bioanal..

[393] Sumner L.W., Amberg A., Barrett D., Beale M.H., Beger R., Daykin C.A., Fan T.W., Fiehn O., Goodacre R., Griffin J.L. (2007). Proposed minimum reporting standards for chemical analysis Chemical Analysis Working Group (CAWG) Metabolomics Standards Initiative (MSI). Metabolomics.

